# Mechanisms affecting exercise ventilatory inefficiency-airflow obstruction relationship in male patients with chronic obstructive pulmonary disease

**DOI:** 10.1186/s12931-020-01463-4

**Published:** 2020-08-06

**Authors:** Ming-Lung Chuang

**Affiliations:** 1 Department of Internal Medicine and Division of Pulmonary Medicine, Chung Shan Medical University Hospital, #110, Section 1, Chien-Kuo North Road, South District, Taichung, 40201 Taiwan, Republic of China; 2 School of Medicine, Chung Shan Medical University, Taichung, 40201 Taiwan, Republic of China

**Keywords:** Incremental exercise test, Obstructive airway disease, Dead space and tidal volume ratio, Ventilatory equivalents for oxygen and CO_2_, Slope for ventilation versus CO_2_ output, Intercept for ventilation versus CO_2_ output

## Abstract

**Background:**

Exercise ventilatory inefficiency is usually defined as high ventilation ($\dot{\mathrm{V}}\mathrm{E}$) versus low CO_2_ output ($\dot{\mathrm{V}}\mathrm{CO}2$). The inefficiency may be lowered when airflow obstruction is severe because $\dot{\mathrm{V}}\mathrm{E}$ cannot be adequately increased in response to exercise. However, the ventilatory inefficiency-airflow obstruction relationship differs to a varying degree. This has been hypothesized to be affected by increased dead space fraction of tidal volume (V_D_/V_T_), acidity, hypoxemia, and hypercapnia.

**Methods:**

A total of 120 male patients with chronic obstructive pulmonary disease were enrolled. Lung function and incremental exercise tests were conducted, and $\dot{\mathrm{V}}\mathrm{E}$ versus $\dot{\mathrm{V}}\mathrm{CO}2$ slope ($\dot{\mathrm{V}}\mathrm{E}/\dot{\mathrm{V}}\mathrm{CO}2\mathrm{S}$) and intercept ($\dot{\mathrm{V}}\mathrm{E}/\dot{\mathrm{V}}\mathrm{CO}2\mathrm{I}$) were obtained by linear regression. Arterial blood gas analysis was also performed in 47 of the participants during exercise tests. V_D_/V_T_ and lactate level were measured.

**Results:**

V_D_/V_Tpeak_ was moderately positively related to $\dot{\mathrm{V}}\mathrm{E}/\dot{\mathrm{V}}\mathrm{CO}2\mathrm{S}$ (*r* = 0.41) and negatively related to forced expired volume in 1 sec % predicted (FEV_1_%) (*r* = − 0.27), and hence the FEV_1_%- $\dot{\mathrm{V}}\mathrm{E}/\dot{\mathrm{V}}\mathrm{CO}2\mathrm{S}$ relationship was paradoxical. The higher the $\dot{\mathrm{V}}\mathrm{E}/\dot{\mathrm{V}}\mathrm{CO}2\mathrm{S}$, the higher the pH and P_a_O_2_, and the lower the P_a_CO_2_ and exercise capacity. $\dot{\mathrm{V}}\mathrm{E}/\dot{\mathrm{V}}\mathrm{CO}2\mathrm{I}$ was marginally related to V_D_/V_Trest_. The higher the $\dot{\mathrm{V}}\mathrm{E}/\dot{\mathrm{V}}\mathrm{CO}2\mathrm{I}$, the higher the inspiratory airflow, work rate, and end-tidal PCO_2peak_.

**Conclusion:**

1) Dead space ventilation perturbs the airflow- $\dot{\mathrm{V}}\mathrm{E}/\dot{\mathrm{V}}\mathrm{CO}2\mathrm{S}$ relationship, 2) increasing ventilation thereby increases $\dot{\mathrm{V}}\mathrm{E}/\dot{\mathrm{V}}\mathrm{CO}2\mathrm{S}$ to maintain biological homeostasis, and 3) the physiology- $\dot{\mathrm{V}}\mathrm{E}/\dot{\mathrm{V}}\mathrm{CO}2\mathrm{S}$- $\dot{\mathrm{V}}\mathrm{E}/\dot{\mathrm{V}}\mathrm{CO}2\mathrm{I}$ relationships are inconsistent in the current and previous studies.

**Trial Registration:**

MOST 106–2314-B-040-025.

## Background

High ventilatory equivalents for oxygen and CO_2_ ($\dot{\mathrm{V}}\mathrm{E}/\dot{\mathrm{V}}\mathrm{O}2$ and $\dot{\mathrm{V}}\mathrm{E}/\dot{\mathrm{V}}\mathrm{CO}2$) have been shown to be indexes of uneven alveolar ventilation-perfusion ratio ($\dot{\mathrm{V}}\mathrm{A}/\dot{\mathrm{Q}}$) [1] and markers of ventilation inefficiency caused by both heart and lung diseases [2]. The $\dot{\mathrm{V}}\mathrm{E}/\dot{\mathrm{V}}\mathrm{CO}2$ slope ($\dot{\mathrm{V}}\mathrm{E}/\dot{\mathrm{V}}\mathrm{CO}2\mathrm{S}$) is elevated in dyspneic patients and can differentiate congestive heart failure (CHF) from chronic obstructive pulmonary disease (COPD) with exercise impairment [3]. $\dot{\mathrm{V}}\mathrm{E}/\dot{\mathrm{V}}\mathrm{CO}2\mathrm{S}$ has also been shown to be a marker of the severity and prognosis of CHF [4, 5] and an indicator of treatment response [6, 7], even though it cannot reflect the treatment effect in patients with CHF of different severity [8].

Compared to $\dot{\mathrm{V}}\mathrm{E}/\dot{\mathrm{V}}\mathrm{CO}2$ ratio ($\dot{\mathrm{V}}\mathrm{E}/\dot{\mathrm{V}}\mathrm{CO}2\mathrm{R}$) in COPD, $\dot{\mathrm{V}}\mathrm{E}/\dot{\mathrm{V}}\mathrm{CO}2$ intercept ($\dot{\mathrm{V}}\mathrm{E}/\dot{\mathrm{V}}\mathrm{CO}2\mathrm{I}$) i.e. dead space ventilation [9, 10], has been shown to be a better indicator of exertional ventilatory inefficiency and unfavorable patient outcomes i.e. mechanical constraint, pulmonary gas exchange, exertional dyspnea, and exercise intolerance [11]. In patients with COPD, $\dot{\mathrm{V}}\mathrm{E}/\dot{\mathrm{V}}\mathrm{CO}2\mathrm{S}$ is negatively related to $\dot{\mathrm{V}}\mathrm{E}/\dot{\mathrm{V}}\mathrm{CO}2\mathrm{I}$ and decreases when airflow obstruction [11] and emphysema are severe [12]. However, in patients with COPD, the relationship between $\dot{\mathrm{V}}\mathrm{E}/\dot{\mathrm{V}}\mathrm{CO}2\mathrm{S}$ and forced expired volume in one s % predicted (FEV_1_%) is weak [3, 11, 13], although it is slightly better when Global Initiative for Chronic Lung Disease (GOLD) staging is used to grade the severity [11]. Similarly, in patients with CHF the slope is increased, however it decreases when the patients have airflow limitation [12] or when an external dead space is large enough to hamper $\dot{\mathrm{V}}\mathrm{E}$ compensation for hypercapnia [9].

Several mechanisms to explain overlapping $\dot{\mathrm{V}}\mathrm{E}/\dot{\mathrm{V}}\mathrm{CO}2\mathrm{I}$ values across GOLD stage I to IV have been proposed [11]. These mechanisms include various afferent information from working limbs [14], peripheral chemoreceptors [15], pulmonary artery pressure, and V_D_/V_T_. However, no data or references have been reported for the last two factors [11].

In COPD, the lower the FEV_1_%, the lower the $\dot{\mathrm{V}}\mathrm{E}/\dot{\mathrm{V}}\mathrm{CO}2\mathrm{S}$ [11, 13], and the lower the FEV_1_%, the larger the V_D_/V_T_ [16, 17]. In contrast, the larger the V_D_/V_T_, the higher the $\dot{\mathrm{V}}\mathrm{E}/\dot{\mathrm{V}}\mathrm{CO}2\mathrm{S}$ [1, 18]. In this context, $\dot{\mathrm{V}}\mathrm{E}/\dot{\mathrm{V}}\mathrm{CO}2\mathrm{S}$ may be high or low at a given FEV_1_%. Hence, we hypothesized that the positive but weak relationship between $\dot{\mathrm{V}}\mathrm{E}/\dot{\mathrm{V}}\mathrm{CO}2\mathrm{S}$ and FEV_1_% may be influenced by V_D_/V_T_. We also evaluated other factors that may influence the relationship including hypoxemia and/or metabolic and/or respiratory acidity. This study aimed to elucidate the mechanisms underpinning the unclear relationship between FEV_1_% and $\dot{\mathrm{V}}\mathrm{E}/\dot{\mathrm{V}}\mathrm{CO}2\mathrm{S}$ and between $\dot{\mathrm{V}}\mathrm{E}/\dot{\mathrm{V}}\mathrm{CO}2\mathrm{S}$ and exercise biological homeostasis.

## Methods

### Study design

We conducted an observational cross-sectional study on incremental maximal exercise in subjects with COPD at our institution. To obtain invasive measurement data, arterial catheterization was established for blood gas sampling in a subgroup of the participants. Each subject signed informed consent before entering the study. The local Institutional Review Board of our institutions (CS19014) approved this study. This study was conducted in compliance with the Declaration of Helsinki.

### Subjects

We enrolled subjects aged ≥40 years with COPD but without any chronic diseases including uncontrolled diabetes mellitus, uncontrolled hypertension, anemia (hemoglobin < 13 g·dL^− 1^ in males), and no acute illnesses in the recent 1 month. The FEV_1_/forced vital capacity (FVC) was < 0.7 [19]. The diagnosis of COPD was made by pulmonologists according to the GOLD criteria [19]. All of the participants had to be able and willing to perform the study protocol including a maximal or symptom-limited cardiopulmonary exercise test (CPET). All of the participants were regularly followed-up at our pulmonary outpatient clinics and received optimized and individually tailored drug treatment, and they all had a stable clinical condition for at least 1 month.

We excluded subjects with a body mass index ≤18 kg·m^− 2^ or ≥ 32 kg·m^− 2^ and those with laboratory findings of hematological, metabolic or neuromuscular diseases, as these factors may confound exercise performance. Subjects with coexisting heart failure with/without documented pulmonary embolism, primary valvular heart disease, pulmonary artery hypertension, pericardial disease, exercise-induced angina, ST changes, and severe arrhythmias were also excluded. As few female subjects meet the criteria of COPD in Taiwan [20], they were not included in this study. We also excluded those who had contraindications to perform the exercise test and those who were participating in exercise training. However, recreational activity was allowed.

### Measurements

#### Demographic and anthropometric data

Age, height, weight, body mass index, and cigarette consumption were recorded.

#### Functional daily activity

The oxygen-cost diagram (OCD) was used to evaluate the participants’ functional activity. The participants were asked to indicate a point on an OCD, a 10-cm long vertical line with everyday activities listed alongside the line, above which breathlessness limited them [21]. The distance from zero was measured and scored.

#### Pulmonary function testing

Cigarette smoking, drinking coffee, tea, or alcohol, and taking medications were not permitted 24 h before any test. Bronchodilators were not administered within 3 h for short-acting beta agonists and 12 h for long-acting beta agonists before the tests [22, 23]. FEV_1_, FVC, total lung capacity (TLC), residual volume (RV), and diffusing capacity for carbon monoxide (D_L_CO) were measured using spirometry, body plethysmography and the single-breath technique (MasterScreen™ Body, Carefusion, Wuerzburg, Germany), respectively in accordance with the currently recommended standards [24, 25]. The best of three technically satisfactory readings was used [24, 26, 27]. All of the spirometry data were obtained before and after inhaling 400 μg of fenoterol HCl. Post-dose measurements were performed 15 min after inhalation. Static lung volume data and D_L_CO data were obtained before inhaling fenoterol. For details, please refer to reference [22].

#### CPET

Each subject completed pulmonary gas exchange measured at rest and during exercise on the different days within 1 month after lung function test. Short-acting and long-acting beta bronchodilators were withheld 4–6 h and ≥ 12 h before the test, respectively. Gas exchange equipment including a face mask connected to a turbine pneumotachograph was used to measured $\dot{\mathrm{V}}\mathrm{O}2$ (mL/min), CO_2_ output ($\dot{\mathrm{V}}\mathrm{CO}2$) (mL/min), minute ventilation ($\dot{\mathrm{V}}\mathrm{E}$) (L/min), tidal volume (V_T_) (L), breathing frequency (b/min), and end-tidal PCO_2_ (P_ET_CO_2_) (mm Hg) breath-by-breath (MasterScreen CPX™, Carefusion, Wuerzburg, Germany), and then the data were averaged and reported at 15-s intervals of each stage using a computer. For each test, 12-lead electrocardiograms were recorded, pulse oximetry was used to record arterial oxyhemoglobin saturation (S_P_O_2_, %), and a sphygmomanometer was used to measure blood pressure every 2 min. An electromagnetically braked cycle ergometer (Lode, Groningen, the Netherlands) was used to adjust workload via a computer. The exercise test protocol was a 2-min period of rest followed by 2-min period of unloaded exercise, followed by ramp-pattern loaded exercise with a workload per stage selected according to the oxygen-cost diagram so that the loaded exercise could be completed within 10 ± 2 min of each participant reaching the limit of symptoms [28]. During each test, a pedaling frequency of 60 rpm was maintained with the aid of a visual pedal rate indicator. Calibrations of the turbine pneumotachograph were performed using a 3-L syringe before each test. The O2 and CO2 analyzers were calibrated with standard gases.

#### Calculation of $\dot{V}E/\dot{V} CO2S$ and $\dot{V}E/\dot{V} CO2R$

Linear regression was used to quantify the relationship between $\dot{\mathrm{V}}\mathrm{E}$ and $\dot{\mathrm{V}}\mathrm{CO}2$ to obtain $\dot{\mathrm{V}}\mathrm{E}/\dot{\mathrm{V}}\mathrm{CO}2\mathrm{S}$ and $\dot{\mathrm{V}}\mathrm{E}/\dot{\mathrm{V}}\mathrm{CO}2\mathrm{I}$. For linear regression, data of the entire loaded exercise [5] were used if the respiratory or ventilatory compensation point (RCP or VCP) [1, 29] were not identified by P_ET_CO_2_ curve; data below the RCP were used if the RCP or VCP was identified. P_ET_CO_2_ curve reveals slow increase from start of exercise to anaerobic threshold and is then relatively stable during isocapneic buffering period. After the period, P_ET_CO_2_ starts to decrease where RCP is defined. To be noted, RCP was reported in four of 16 subjects with pulmonary emphysema in a previous study [12]. $\dot{\mathrm{V}}\mathrm{E}/\dot{\mathrm{V}}\mathrm{CO}2\mathrm{R}$ was directly calculated. $\dot{\mathrm{V}}\mathrm{E}/\dot{\mathrm{V}}\mathrm{CO}2$ nadir ($\dot{\mathrm{V}}\mathrm{E}/\dot{\mathrm{V}}\mathrm{CO}2\mathrm{N}$) was the lowest value of $\dot{\mathrm{V}}\mathrm{E}/\dot{\mathrm{V}}\mathrm{CO}2\mathrm{R}$ during loaded exercise period [30].

#### V_D_/V_T_ measurement

Brachial artery catheterization was established and blood samples were drawn and heparinized in a subgroup of the participants at rest and at the last 15 s of every minute during loaded exercise and at peak exercise. The sample was immediately placed on ice and then analyzed for pH, PCO_2_, and PO_2_ with body temperature correction (model 278, CIBA-Corning, Medfield, MA, USA). The V_D_/V_T_ was calculated using a standard formula as follows [31].

1 $$ {V}_D/{V}_T=\left({P}_a{CO}_2-P\ \overline{\mathrm{E}}\ {CO}_2\right)/{P}_a{CO}_2-{V}_Dm/{V}_T $$

where P $\overline{\mathrm{E}}$ CO_2_ = $\dot{\mathrm{V}}\mathrm{CO}2/\dot{\mathrm{V}}\mathrm{E}$ × (P_B_ - 47 mmHg) and PB was barometric pressure measured daily and V_D_m was the dead space of mouth piece and pneumotachograph as the manufacture reported.

### Statistical analysis

Data were summarized as mean ± standard deviation. Comparisons between two groups were performed using two-sample t test. Pearson’s or Spearman’s correlation coefficients were used when appropriate for quantifying the pair-wise relationships among the interested continuous variables. Statistical significance was set at *p ≤* 0.05.

Marginal statistical significance was set at 0.05 < *p* < 0.1.

## Results

**Fig. 1:**
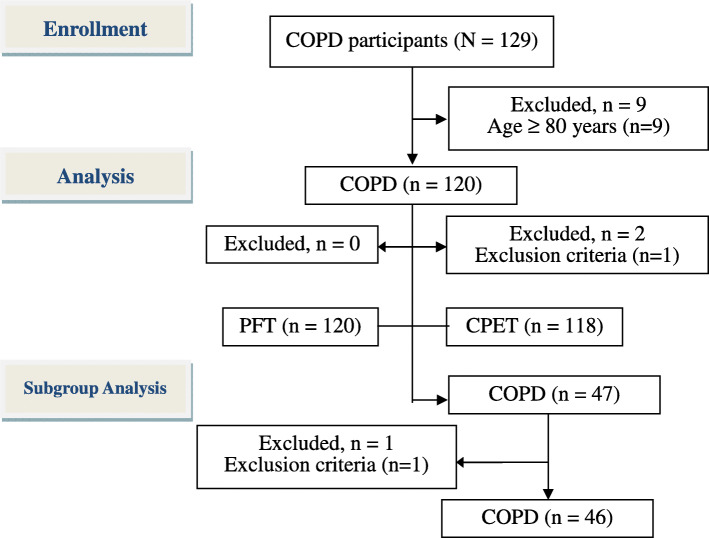
Flow diagram. A total of 120 male patients with chronic obstructive pulmonary disease were enrolled after excluding nine subjects aged 80 ≥ years. 118 participants completed the lung function test and 46 patients, the subgroup, completed arterial blood gas analysis during exercise

A total of 120 male subjects with COPD aged 67.0 ± 6.8 years were enrolled after excluding nine subjects aged ≥80 years (Fig. 1 and Table 1). Most of the participants had moderate to severe disease severity. Overall, 118 subjects completed the exercise test after excluding two who had poor motivation (Table 1). In the entire group and its subgroup of patients who underwent blood gas sampling, $\dot{\mathrm{V}}\mathrm{E}/\dot{\mathrm{V}}\mathrm{CO}2\mathrm{S}$ and $\dot{\mathrm{V}}\mathrm{E}/\dot{\mathrm{V}}\mathrm{CO}2\mathrm{I}$ were moderately negatively related (Table 2, *r* = − 0.40 - − 0.44, *p* < 0.001 - < 0.0001). The relationships between $\dot{\mathrm{V}}\mathrm{E}/\dot{\mathrm{V}}\mathrm{CO}2\mathrm{S}$ and the pulmonary physiology variables of interest were similar to some extent between the entire group and the subgroup of patients who underwent blood gas sampling (Table 2).

**Table 1:** Subjects’ characteristics, lung function, and exercise data (*n* = 120) versus the subgroup data (*n* = 47)

*N* =	Total		Subgroup		
	Mean	SD	Mean	SD	T test
	120		47		
Age, year	67.0	6.8	65.3	5.7	NS
Height, cm	164.8	5.7	165.1	6.4	NS
Weight, kg	62.4	9.4	60.8	11.4	NS
Body mass index, kg/m^2^	23.0	3.1	22.2	3.6	NS
Smoke, pack-year	51.3	28.1	41.4	19.3	0.01
Oxygen-cost diagram, cm	7.1	1.3	7.0	1.4	NS
Total lung capacity, TLCpred%	1.15	0.23	1.34	0.21	< 0.0001
Residual volume/TLC	0.56	0.10	0.58	0.09	NS
D_L_CO%	0.76	0.24	0.69	0.22	NS
Forced vital capacity, FVCpred%	0.83	0.20	0.81	0.21	NS
FEV_1_pred%	0.57	0.18	0.50	0.19	0.06
GOLD I-IV, *n*=	10, 68, 33, 9		3, 19, 19, 6		
FEV_1_/FVC	0.53	0.12	0.49	0.13	0.1
Heart Rate_peak_%	0.82	0.11	0.81	0.12	NS
Oxygen uptake, VO_2peak_%,	0.69	0.20	0.69	0.21	NS
Respiratory exchange ratio_peak_	1.05	0.10	1.05	0.10	NS
Work_peak_%	0.75	0.26	0.68	0.30	NS
O_2_Pulse_peak_%	0.83	0.22	0.85	0.23	NS
Minute ventilation, V_Epeak_, L/min,	43.6	13.1	38.6	12.3	0.02
V_E peak_/MVV	1.00	0.30	1.16	0.36	< 0.01
V_E_/VCO_2nadir_	38.6	7.8	35.0	6.9	< 0.01
V_E_/VCO_2_ Slope	33.7	7.5	29.9	5.7	< 0.001
V_E_/VCO_2_ Intercept	5.2	1.8	5.2	1.6	NS
S_P_O_2peak_, %	92.2	5.4	91.0	5.8	NS
Tidal volume, V_Tpeak_/TLC	0.22	0.06	0.19	0.05	< 0.01
V_T_/Inspiratory time, T_Ipeak_, L/s	1.70	0.50	1.52	0.46	0.04
Breathing frequency_peak_, b/min	33.5	6.1	32.6	5.9	NS
Breathing cycle time, T_tot peak_, s	1.85	0.33	1.89	0.31	NS
T_Ipeak_, s	0.78	0.16	0.78	0.13	NS
RSBI_peak_, b/L	27.8	11.3	30.5	13.9	NS

*D*_*L*_*CO* diffusing capacity for carbon monoxide, *FEV*_*1*_ forced expired volume in 1 s, *GOLD* global initiatives for chronic obstructive lung disease, *O*_*2*_*Pulse* V’O_2_/heart rate, *MVV* maximal voluntary ventilation, *S*_*P*_*O*_*2*_ oxyhemoglobin saturation measured with pulse oximetry, *s* second, *RSBI* rapid shallow breathing index = breathing frequency/tidal volume

**Table 2:** Summary of correlation (r) of $\dot{\mathrm{V}}\mathrm{E}/\dot{\mathrm{V}}\mathrm{CO}2$ slope ($\dot{\mathrm{V}}\mathrm{E}/\dot{\mathrm{V}}\mathrm{CO}2\mathrm{S}$) and its intercept ($\dot{\mathrm{V}}\mathrm{E}/\dot{\mathrm{V}}\mathrm{CO}2\mathrm{I}$) with pulmonary physiology

r	Slope		Intercept	
*N* =	118	46	118	46
Intercept	-0.44^†^	−0.40^**^	1	1
Expiration				
FEV_1_%	0.20^*^	0.42^**^	−0.09	−0.12
FEV_1_/VC	0.27^**^	0.15	0.02	−0.02
GOLD	−0.26^**^	−0.44^**^	0.08	0.11
Inspiration				
V_T_/T_Ipeak_	0.20^¶^	0.03	0.22^*^	0.30^*^
Volume excursion/dynamic hyperinflation:				
V_Tpeak_/FEV_1_	−0.15	−0.32^*^	0.14	0.18
V_Tpeak_/IC	−0.15	−0.05	0.15	−0.00
V_Tpeak_/VC	0.01	−0.04	0.02	−0.04
V_Tpeak_/TLC	−0.01	−0.06	0.10	0.13
Gas exchange:				
S_P_O_2peak_	0.32^***^	0.50^***^	0.03	−0.19
P_ET_CO_2peak-rest_	–	− 0.62^†^	–	0.53^***^
V_D_/V_Trest_	–	0.03	–	0.28^¶^
V_D_/V_Tpeak_	–	0.41^**^	–	−0.23
Exercise capacity				
$\dot{\mathrm{V}}\mathrm{O}2$_peak_^θ^	−0.33^***^	− 0.27^¶^	0.28^**^	0.27^¶^
Work_peak_^θ^	−0.1	−0.3^*^	0.30^***^	0.43^**^

*Abbreviations*: *FEV*_*1*_ forced expired volume in 1 s, *VC* vital capacity, *GOLD* stage of global initiatives for chronic obstructive lung disease, *V*_*T*_*/T*_*Ipeak*_ the ratio of tidal volume and inspiratory time in second indicating mean inspiratory flow, *IC* inspiratory capacity, *TLC* total lung capacity, *S*_*P*_*O*_*2*_ oxyhemoglobin measured by pulse oximetry, *P*_*ET*_*CO*_*2*_ end-tidal CO_2_ pressure, $\dot{\mathrm{V}}\mathrm{O}2$ oxygen uptake

^θ^% of predicted maximum

−-: not available

^¶^ 0.05 < *p* < 0.1

^*^ < 0.05

^**^ < 0.01

^***^ ≤ 0.001

^†^< 0.0001

$\dot{V}E/\dot{V} CO2S$ versus *Pulmonary Physiology and Exercise Capacity*. $\dot{\mathrm{V}}\mathrm{E}/\dot{\mathrm{V}}\mathrm{CO}2\mathrm{S}$ was related to a varying degree to expiratory flow (*r* = 0.20–0.42, *p* < 0.05 - < 0.01), and marginally related to inspiratory flow. $\dot{\mathrm{V}}\mathrm{E}/\dot{\mathrm{V}}\mathrm{CO}2\mathrm{S}$ was not related to any of the volume excursion variables at peak exercise except for V_T_/FEV_1_ in the subgroup analysis (Table 2, *r* = − 0.32, *p* < 0.05). $\dot{\mathrm{V}}\mathrm{E}/\dot{\mathrm{V}}\mathrm{CO}2\mathrm{S}$ was positively related to an increase in S_P_O_2_ (*r* = 0.32–0.50). $\dot{\mathrm{V}}\mathrm{E}/\dot{\mathrm{V}}\mathrm{CO}2\mathrm{S}$ was mildly negatively related to $\dot{\mathrm{V}}\mathrm{O}2$_peak_% (*r* = − 0.27 - -0.33). In the subgroup of patients who underwent blood gas sampling, at peak exercise, $\dot{\mathrm{V}}\mathrm{E}/\dot{\mathrm{V}}\mathrm{CO}2\mathrm{S}$ was moderately positively related to pH and P_a_O_2_ (Table 3, *r* = 0.40–0.53), and strongly negatively related to P_a_CO_2_ and P_ET_CO_2_ (Tables 2 and 3, *r* = − 0.60 - -0.62).

**Table 3:**
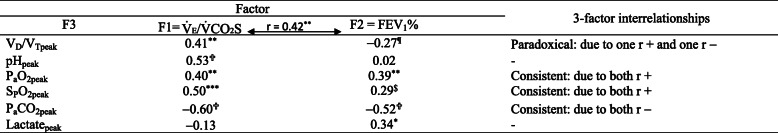
Three-factor interrelationships in 46 subjects with COPD

**Fig. 2:**
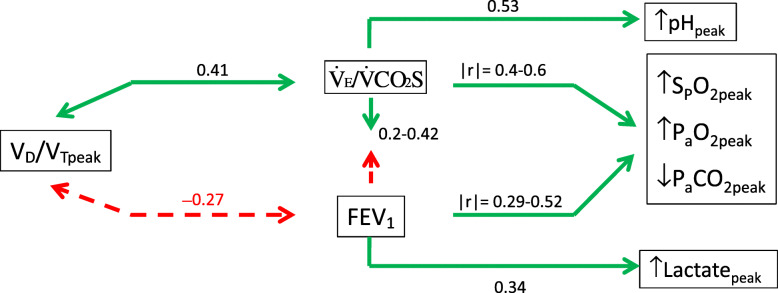
Flow chart showing the deductive mechanism of exercise ventilatory inefficiency and biological homeostasis. V_D_/V_T_: dead space fraction of tidal volume, $\dot{\mathrm{V}}\mathrm{E}/\dot{\mathrm{V}}\mathrm{CO}2\mathrm{S}$: minute ventilation versus CO_2_ output slope, FEV_1_: forced expired volume in one s, S_P_O_2peak_: oxyhemoglobin saturation measured by pulse oximetry at peak exercise, P_a_O_2_: arterial partial pressure of O_2_, P_a_CO_2_: arterial partial pressure of CO_2_. Solid line with two-direction arrowheads: positive correlation, dashed line with two-direction arrowheads: negative correlation. Solid line with a single direction arrowhead: positively inducing, dashed line with a single direction arrowhead: negatively inducing

In the subgroup of patients who underwent blood gas sampling, with regards to pulmonary physiology variables, V_D_/V_Tpeak_ was moderately positively related to $\dot{\mathrm{V}}\mathrm{E}/\dot{\mathrm{V}}\mathrm{CO}2\mathrm{S}$, and marginally negatively related to FEV_1_% (Table 2 and Fig. 2, *r* = − 0.27, *p* = 0.08).

$\dot{V}E/\dot{V} CO2I$ versus *Pulmonary Physiology and Exercise Capacity*. $\dot{\mathrm{V}}\mathrm{E}/\dot{\mathrm{V}}\mathrm{CO}2\mathrm{I}$ was mildly related to inspiratory flow (*r* = 0.22–0.30, *p* < 0.05), marginally to mildly related to $\dot{\mathrm{V}}\mathrm{O}2$_peak_% (Table 2, *r* = 0.27–0.28) and mildly to moderately related to Work_peak_% (Table 2, *r* = 0.30–0.43), but not to expiratory flow or all volume excursion variables.

In the subgroup of patients who underwent blood gas sampling, $\dot{\mathrm{V}}\mathrm{E}/\dot{\mathrm{V}}\mathrm{CO}2\mathrm{I}$ was moderately related to an increase in P_ET_CO_2_ (Table 2, *r* = 0.53) and marginally related to V_D_/V_Trest_ (*r* = 0.28, *p* = 0.08), but not to V_D_/V_Tpeak_.

## Discussion

The main findings of this study confirm that in male subjects with COPD, $\dot{\mathrm{V}}\mathrm{E}/\dot{\mathrm{V}}\mathrm{CO}2\mathrm{S}$ was correlated to a varying degree with FEV_1_% and GOLD stage. We further found that V_D_/V_Tpeak_ was the main cause of the relationships (Fig. 2). A high $\dot{\mathrm{V}}\mathrm{E}/\dot{\mathrm{V}}\mathrm{CO}2\mathrm{S}$ improved arterial pH, PO_2_, and PCO_2_, but was not caused by these factors. The findings support our hypothesis. Additionally, $\dot{\mathrm{V}}\mathrm{E}/\dot{\mathrm{V}}\mathrm{CO}2\mathrm{I}$ was marginally related to dead space at rest and $\dot{\mathrm{V}}\mathrm{O}2\mathrm{peak}$ and significantly related to increases in inspiratory airflow, P_a_CO_2_, and work rate.

$\dot{V}E/\dot{V} CO2S$ versus *Pulmonary Physiology of COPD*. The results revealed that expiratory airflow graded by FEV_1_%, GOLD stage, and FEV_1_/VC was related to $\dot{\mathrm{V}}\mathrm{E}/\dot{\mathrm{V}}\mathrm{CO}2\mathrm{S}$ to a varying degree (Fig. 2 and Table 2, |r| = 0.20–0.44). This is in line with previous reports that in patients with heart and lung diseases, severe airflow impairment may limit $\dot{\mathrm{V}}\mathrm{E}/\dot{\mathrm{V}}\mathrm{CO}2\mathrm{S}$ to compensate for metabolic acidosis during heavy exercise [3, 9, 11, 12]. However, this notion is not consistent with the study by Teopompi et al., who reported that $\dot{\mathrm{V}}\mathrm{E}/\dot{\mathrm{V}}\mathrm{CO}2\mathrm{S}$ and FEV_1_% were not related (Supplementary Table) [13], although the role of inspiratory muscles was not considered. With regards to the tension time index of ventilatory muscle mechanics in normal healthy people and those with a disease, the inspiratory muscles may adapt to a level below or within the critical zone to sustain breathing in various conditions [32, 33]. As the mechanical load increases to a level which the inspiratory muscles can no longer tolerate, alveolar hypoventilation develops and the P_a_CO_2_ point may be reset [34]. However, in the current study, mean inspiratory airflow was marginally related to $\dot{\mathrm{V}}\mathrm{E}/\dot{\mathrm{V}}\mathrm{CO}2\mathrm{S}$ in the entire group and not significantly related to $\dot{\mathrm{V}}\mathrm{E}/\dot{\mathrm{V}}\mathrm{CO}2\mathrm{S}$ in the subgroup, suggesting that mean inspiratory airflow was not sensitive enough to be related to $\dot{\mathrm{V}}\mathrm{E}/\dot{\mathrm{V}}\mathrm{CO}2\mathrm{S}$.

However, expiratory airflow was related to $\dot{\mathrm{V}}\mathrm{E}/\dot{\mathrm{V}}\mathrm{CO}2\mathrm{S}$ to a varying degree, which may be explained by V_D_/V_T_. In the current study, V_D_/V_Tpeak_ was positively related to $\dot{\mathrm{V}}\mathrm{E}/\dot{\mathrm{V}}\mathrm{CO}2\mathrm{S}$, similar to previous reports which used $\dot{\mathrm{V}}\mathrm{E}/\dot{\mathrm{V}}\mathrm{CO}2\mathrm{R}$ ranging from 31 to 40 in parallel with a V_D_/V_T_ ratio ranging from 0.37 to 0.49 [16]. Combining the positive V_D_/V_Tpeak_- $\dot{\mathrm{V}}\mathrm{E}/\dot{\mathrm{V}}\mathrm{CO}2\mathrm{S}$ relationship with the positive FEV_1_%- $\dot{\mathrm{V}}\mathrm{E}/\dot{\mathrm{V}}\mathrm{CO}2\mathrm{S}$ relationship, it can be deduced that a high V_D_/V_Tpeak_ and a high FEV_1_% together may synergistically amplify $\dot{\mathrm{V}}\mathrm{E}/\dot{\mathrm{V}}\mathrm{CO}2\mathrm{S}$ (Fig. 2). However, FEV_1_% and V_D_/V_Tpeak_ were negatively related in this study (*r* = − 0.27) and in a previous report (*r* = − 0.377) [17]. As a result, the relationship between FEV_1_% and $\dot{\mathrm{V}}\mathrm{E}/\dot{\mathrm{V}}\mathrm{CO}2\mathrm{S}$ was perturbed [3, 11, 13]. Hence, the relationship between V_D_/V_Tpeak_ and $\dot{\mathrm{V}}\mathrm{E}/\dot{\mathrm{V}}\mathrm{CO}2\mathrm{S}$ may also have been perturbed (Fig. 2 and Table 3).

Nevertheless, the high V_D_/V_T_ was also biphasic, i.e. it caused an increase or decrease in $\dot{\mathrm{V}}\mathrm{E}$ at a given level of metabolism. An appropriately high V_D_/V_T_ may increase $\dot{\mathrm{V}}\mathrm{E}$ to maintain arterial isocapnia. However, Poon and Tin [35] and Gargiuro et al. [9] reported that excessive mechanical constraints may occur in patients with CHF when external dead space volume is loaded to an inappropriate extent. The biphasic effect of high V_D_/V_Tpeak_ on $\dot{\mathrm{V}}\mathrm{E}$ may further modify the $\dot{\mathrm{V}}\mathrm{E}/\dot{\mathrm{V}}\mathrm{CO}2\mathrm{S}$-FEV_1_% relationship.

At peak exercise, the more severe the airflow obstruction and emphysema, the lower the $\dot{\mathrm{V}}\mathrm{E}/\dot{\mathrm{V}}\mathrm{CO}2\mathrm{S}$ [3, 11, 12]. Although Paolotti et al. [12] agreed with this notion, they proposed another two hypotheses: (1) an improvement in ventilatory efficiency during exercise due to reduced physiological dead space; (2) a higher arterial CO_2_ (PaCO_2_) set-point, as they found that the hypercapnia was related to emphysema. In this study, the increase in $\dot{\mathrm{V}}\mathrm{E}/\dot{\mathrm{V}}\mathrm{CO}2\mathrm{S}$ at peak exercise was related to an increase in V_D_/V_T_ but not to a decrease in V_D_/V_T_. A higher P_a_CO_2_ point was not reset; instead, a lower P_a_CO_2_ level developed. Notably, only 10 subjects had arterial blood gas data during exercise in their study, and the formula for V_D_/V_T_ did not subtract apparatus V_D_ [12], which was addressed by Wasserman et al. and Sun et al. [2, 30]. A high FEV_1_% is associated with a high $\dot{\mathrm{V}}\mathrm{E}$; a high $\dot{\mathrm{V}}\mathrm{E}$ is associated with a high $\dot{\mathrm{V}}\mathrm{E}/\dot{\mathrm{V}}\mathrm{CO}2\mathrm{S}$; a high $\dot{\mathrm{V}}\mathrm{E}/\dot{\mathrm{V}}\mathrm{CO}2\mathrm{S}$ is associated with a high pH and P_a_O_2_, and a low P_a_CO_2_ (Fig. 2). In other words, this also suggests that mechanical constraints may limit the increase in $\dot{\mathrm{V}}\mathrm{E}$ during exercise with a negative influence on gas exchange values at peak exercise (i.e. P_a_O_2_ and S_P_O_2_ decrease, P_a_CO_2_ increase).

Interestingly, $\dot{\mathrm{V}}\mathrm{E}/\dot{\mathrm{V}}\mathrm{CO}2\mathrm{S}$ was highly negatively related to emphysema (*r* = − 0.77, *p* < 0.001) [12] in Paolotti et al’s study and in the current study as represented by V_Tpeak_/FEV_1_ as the emphysema factor [13] (Table 2), whereas it was moderately positively related to V_D_/V_Tpeak_ in the current study and in another report [16]. In this context, it can be deduced that emphysema may be inversely related to V_D_/V_Tpeak_. However, Paoletti et al. reported that when emphysema was measured by high resolution computed tomography, the FEV_1_% and V_D_/V_Tpeak-rest_ were weakly related to the emphysema extent [12, 36]. When emphysema was evaluated by pathology, the feature of loss of alveolar attachments was related to high $\dot{\mathrm{V}}\mathrm{D}$ and V_D_/V_T_ [37] and low FEV_1_% [17].

Volume excursion at peak exercise i.e. V_T_/IC and V_T_/VC and V_T_/FEV_1_ (emphysema factor) [13] and dynamic hyperinflation (DH) as represented by EELV_peak_ /TLC [11] have been reported to be mildly to moderately negatively related to $\dot{\mathrm{V}}\mathrm{E}/\dot{\mathrm{V}}\mathrm{CO}2\mathrm{S}$ in the literature (Supplementary Table, *r* = − 0.31 - -0.35 and − 0.48 - -0.60). However, in the current study, even though none of the markers of volume excursion and DH as represented by V_T_/TLC [38, 39] were related to $\dot{\mathrm{V}}\mathrm{E}/\dot{\mathrm{V}}\mathrm{CO}2\mathrm{S}$, the emphysema factor was mildly negatively related to $\dot{\mathrm{V}}\mathrm{E}/\dot{\mathrm{V}}\mathrm{CO}2\mathrm{S}$ (*r* = − 0.32).

$\dot{V}E/\dot{V} CO2I$ versus *Pulmonary Physiology*. In patients with heart failure and normal subjects with or without external V_D_ at rest and during exercise, $\dot{\mathrm{V}}\mathrm{E}/\dot{\mathrm{V}}\mathrm{CO}2\mathrm{I}$ is assumed to be $\dot{\mathrm{V}}\mathrm{D}$ when $\dot{\mathrm{V}}\mathrm{CO}2$ is zero [9, 40]. However, our findings may challenge this notion, as $\dot{\mathrm{V}}\mathrm{E}/\dot{\mathrm{V}}\mathrm{CO}2\mathrm{I}$ was not significantly related to V_D_/V_Trest_ or V_D_/V_Tpeak_ (Table 2). Other studies have also not supported that $\dot{\mathrm{V}}\mathrm{E}/\dot{\mathrm{V}}\mathrm{CO}2\mathrm{I}$ is an index of $\dot{\mathrm{V}}\mathrm{D}$. The $\dot{\mathrm{V}}\mathrm{E}/\dot{\mathrm{V}}\mathrm{CO}2\mathrm{I}$ has been reported to be ≤0 L in more than 10% of subjects in previous reports [3, 29] even though other studies have reported no patients with ≤0 L (0.9–9.9 L) [13]. In normal subjects, Sun et al. reported a $\dot{\mathrm{V}}\mathrm{E}/\dot{\mathrm{V}}\mathrm{CO}2\mathrm{I}$ value of 11.7 L/min [30]. In patients with heart failure, Gargiulo et al. reported that the average of V_D_ and $\dot{\mathrm{V}}\mathrm{E}/\dot{\mathrm{V}}\mathrm{CO}2\mathrm{I}$ at rest was 0.3–0.5 L ± 0.2 L, with a V_T_ of 0.38 ± 0.08 L [9]. These values are too large to be biological plausible for V_D_ and $\dot{\mathrm{V}}\mathrm{D}$ in their study [9]. Nevertheless, the apparatus V_D_ was also not subtracted from the physiological V_D_ when calculating V_D_/V_T_ [9]. In this context, despite an increase in P_ET_CO_2_ being moderately related to $\dot{\mathrm{V}}\mathrm{E}/\dot{\mathrm{V}}\mathrm{CO}2\mathrm{I}$ in the current study and to $\dot{\mathrm{V}}\mathrm{E}/\dot{\mathrm{V}}\mathrm{CO}2\mathrm{S}$ in Paoletti et al’s report [12], whether or not $\dot{\mathrm{V}}\mathrm{E}/\dot{\mathrm{V}}\mathrm{CO}2\mathrm{I}$ reflects $\dot{\mathrm{V}}\mathrm{D}$ remains unclear.

On the other hand, in the current study, we found that $\dot{\mathrm{V}}\mathrm{E}/\dot{\mathrm{V}}\mathrm{CO}2\mathrm{I}$ was mildly related to inspiratory flow rather than FEV_1_% (Table 2). The loss of alveolar attachments is a feature of emphysema with high $\dot{\mathrm{V}}\mathrm{D}$ and V_D_/V_T_ [37] and is usually measured in fully inflated lungs so that expiratory flow obstruction cannot sufficiently reflect the condition, and thus its severity can be underestimated [41]. However, Teopompi et al. reported that $\dot{\mathrm{V}}\mathrm{E}/\dot{\mathrm{V}}\mathrm{CO}2\mathrm{I}$ was moderately negatively related to FEV_1_% and diffusing capacity [13]. Moreover, they reported that the inconsistence in the $\dot{\mathrm{V}}\mathrm{E}/\dot{\mathrm{V}}\mathrm{CO}2\mathrm{I}$-FEV_1_% relationship was attributed to volume excursion constraint which developed during exercise [13], whereas volume excursion constraint was not related to $\dot{\mathrm{V}}\mathrm{E}/\dot{\mathrm{V}}\mathrm{CO}2\mathrm{I}$ or $\dot{\mathrm{V}}\mathrm{E}/\dot{\mathrm{V}}\mathrm{CO}2\mathrm{S}$ in the current study.

In the current study, the relationships between $\dot{\mathrm{V}}\mathrm{E}/\dot{\mathrm{V}}\mathrm{CO}2\mathrm{S}$ and $\dot{\mathrm{V}}\mathrm{O}2$_peak_% and Work_peak_% were negative to a varying extent, which is consistent with the previous reports (Table 2 and Supplementary Table) [3, 11, 13]. However, the relationship between $\dot{\mathrm{V}}\mathrm{E}/\dot{\mathrm{V}}\mathrm{CO}2\mathrm{I}$ and $\dot{\mathrm{V}}\mathrm{O}2$_peak_% in the current study was different to a previous report [11] (Table 2 and Supplementary Table). The reason is unclear. In the current study, V_D_/V_Tpeak_ was simultaneously the opposite of $\dot{\mathrm{V}}\mathrm{E}/\dot{\mathrm{V}}\mathrm{CO}2\mathrm{I}$ and $\dot{\mathrm{V}}\mathrm{O}2$_peak_% (*r* = − 0.23 and − 0.62, respectively) and V_T_/T_Ipeak_ was simultaneously consistent with $\dot{\mathrm{V}}\mathrm{E}/\dot{\mathrm{V}}\mathrm{CO}2\mathrm{I}$ and $\dot{\mathrm{V}}\mathrm{O}2$_peak_% (*r* = 0.22–0.30 and 0.59, respectively). The heterogeneity of the population of this study may also have contributed to the inconsistencies. Further studies are warranted to clarify this issue.

Lastly, an interesting finding was the difference between $\dot{\mathrm{V}}\mathrm{E}/\dot{\mathrm{V}}\mathrm{CO}2\mathrm{R}$ and $\dot{\mathrm{V}}\mathrm{E}/\dot{\mathrm{V}}\mathrm{CO}2\mathrm{S}$ in combination with $\dot{\mathrm{V}}\mathrm{E}/\dot{\mathrm{V}}\mathrm{CO}2\mathrm{I}$. $\dot{\mathrm{V}}\mathrm{E}/\dot{\mathrm{V}}\mathrm{CO}2\mathrm{S}$ and $\dot{\mathrm{V}}\mathrm{E}/\dot{\mathrm{V}}\mathrm{CO}2\mathrm{I}$ have consistently been negatively related to a varying degree both in the current study and in previous studies (Table 2, *r* = − 0.25 - -0.74) [11, 13]. The sum of $\dot{\mathrm{V}}\mathrm{E}/\dot{\mathrm{V}}\mathrm{CO}2\mathrm{S}$ and $\dot{\mathrm{V}}\mathrm{E}/\dot{\mathrm{V}}\mathrm{CO}2\mathrm{I}$ was reported to be close to or closely related to $\dot{\mathrm{V}}\mathrm{E}/\dot{\mathrm{V}}\mathrm{CO}2\mathrm{R}$ in a previous report [11]. In the current study, the sum of the two variables and $\dot{\mathrm{V}}\mathrm{E}/\dot{\mathrm{V}}\mathrm{CO}2\mathrm{R}$ were similar (39.5 ± 7.5 versus 38.6 ± 7.8, *p* = 0.52). The relationship between the sum of $\dot{\mathrm{V}}\mathrm{E}/\dot{\mathrm{V}}\mathrm{CO}2\mathrm{S}$ and $\dot{\mathrm{V}}\mathrm{E}/\dot{\mathrm{V}}\mathrm{CO}2\mathrm{I}$ and $\dot{\mathrm{V}}\mathrm{E}/\dot{\mathrm{V}}\mathrm{CO}2\mathrm{R}$ has been reported to be mathematical [1, 2]. Further mathematical simulation studies on this issue are warranted.

### Study limitations

There are several limitations to this study. First, correlation studies allow researchers to study the relationships between one variable and others, and may not be appropriate to infer a cause and effect. However, it is reasonable to consider that a high V_D_/V_T_ may induce $\dot{\mathrm{V}}\mathrm{E}/\dot{\mathrm{V}}\mathrm{CO}2\mathrm{S}$ rather than to consider that a high $\dot{\mathrm{V}}\mathrm{E}/\dot{\mathrm{V}}\mathrm{CO}2\mathrm{S}$ induces a high V_D_/V_T_. Similarly, a high FEV_1_% may induce a high $\dot{\mathrm{V}}\mathrm{E}/\dot{\mathrm{V}}\mathrm{CO}2\mathrm{S}$ rather than a high $\dot{\mathrm{V}}\mathrm{E}/\dot{\mathrm{V}}\mathrm{CO}2\mathrm{S}$ induces a high FEV1%. Second, the number of cases in this subgroup study was small, and this may have caused insufficient power when performing correlation coefficient analysis on V_D_/V_T_ and the other variables of interest. However, the sample size of 46 achieved a power of 80% to detect a difference between a correlation of 0.4 and the null (no correlation) using a two-sided test with a significance level of 0.05. As the power is related to type II error, a non-significant test results should be interpreted more conservatively. Third, all of the participants in this study were male, so the results cannot be applied to females. As only 4% of patients with COPD are female in Taiwan [20], and as breathing pattern and dead space are different between men and women [42], it would be difficult to enroll a sufficient number of female subjects with COPD to compare the differences between male and female patients with COPD. To calculate $\dot{\mathrm{V}}\mathrm{E}/\dot{\mathrm{V}}\mathrm{CO}2\mathrm{S}$ and $\dot{\mathrm{V}}\mathrm{E}/\dot{\mathrm{V}}\mathrm{CO}2\mathrm{I}$, the methodology to identify VCP or RCP [1, 9, 29] and whether to use the entire loaded exercise data [5] or data below VCP/RCP [2, 3, 11–13] are inconsistent in the literature. Further studies are warranted to clarify these issues.

### Clinical implication

Although airflow obstruction may attenuate the increase in $\dot{\mathrm{V}}\mathrm{E}/\dot{\mathrm{V}}\mathrm{CO}2\mathrm{S}$ during incremental exercise, an increase in dead space ventilation may amplify $\dot{\mathrm{V}}\mathrm{E}/\dot{\mathrm{V}}\mathrm{CO}2\mathrm{S}$ and thus perturb the $\dot{\mathrm{V}}\mathrm{E}/\dot{\mathrm{V}}\mathrm{CO}2\mathrm{S}$ - FEV_1_% relationship. Nevertheless, airflow obstruction is usually accompanied with increased dead space ventilation. Hence, this study reveals the paradoxical relationship among the three factors (i.e. $\dot{\mathrm{V}}\mathrm{E}/\dot{\mathrm{V}}\mathrm{CO}2\mathrm{S}$, airflow obstruction and dead space ventilation). The role of $\dot{\mathrm{V}}\mathrm{E}/\dot{\mathrm{V}}\mathrm{CO}2\mathrm{I}$ as a marker of ventilatory insufficiency in COPD is also questionable. Further studies are warranted to study the clinical applications and importance of exercise $\dot{\mathrm{V}}\mathrm{E}/\dot{\mathrm{V}}\mathrm{CO}2\mathrm{S}$ and $\dot{\mathrm{V}}\mathrm{E}/\dot{\mathrm{V}}\mathrm{CO}2\mathrm{I}$ in patients with COPD.

## Conclusions

Using V_D_/V_T_ measurements, we found that dead space ventilation perturbs the airflow- $\dot{\mathrm{V}}\mathrm{E}/\dot{\mathrm{V}}\mathrm{CO}2\mathrm{S}$ relationship. Increasing ventilation thereby increasing $\dot{\mathrm{V}}\mathrm{E}/\dot{\mathrm{V}}\mathrm{CO}2\mathrm{S}$ may be the cause rather than the effect of maintaining biological homeostasis. The pulmonary physiology- $\dot{\mathrm{V}}\mathrm{E}/\dot{\mathrm{V}}\mathrm{CO}2\mathrm{S}$- $\dot{\mathrm{V}}\mathrm{E}/\dot{\mathrm{V}}\mathrm{CO}2\mathrm{I}$ relationship is inconsistent between the current study and previous studies.

## Supplementary information

**Additional file 1: Supplementary Table**. Summary of correlation (r) of $\dot{\mathrm{V}}\mathrm{E}/\dot{\mathrm{V}}\mathrm{CO}2$ slope ($\dot{\mathrm{V}}\mathrm{E}/\dot{\mathrm{V}}\mathrm{CO}2\mathrm{S}$) and its intercept ($\dot{\mathrm{V}}\mathrm{E}/\dot{\mathrm{V}}\mathrm{CO}2\mathrm{I}$) with pulmonary physiology.

**Additional file 2.**

## Data Availability

Uploaded.

## Abbreviations

$$ \dot{\mathrm{V}}\mathrm{E} $$: Ventilation
$$ \dot{\mathrm{V}}\mathrm{CO}2 $$: CO_2_ output
V_D_/V_T_: Dead space fraction of tidal volume
$$ \dot{\mathrm{V}}\mathrm{E}/\dot{\mathrm{V}}\mathrm{CO}2\mathrm{S} $$: $$ \dot{\mathrm{V}}\mathrm{E} $$ versus $$ \dot{\mathrm{V}}\mathrm{CO}2 $$ slope
$$ \dot{\mathrm{V}}\mathrm{E}/\dot{\mathrm{V}}\mathrm{CO}2\mathrm{I} $$: $$ \dot{\mathrm{V}}\mathrm{E} $$ versus $$ \dot{\mathrm{V}}\mathrm{CO}2 $$ intercept
FEV_1_%: Forced expired volume in one s % predicted
CHF: Congestive heart failure
COPD: Chronic obstructive pulmonary disease
$$ \dot{\mathrm{V}}\mathrm{E}/\dot{\mathrm{V}}\mathrm{CO}2\mathrm{R} $$: $$ \dot{\mathrm{V}}\mathrm{E}/\dot{\mathrm{V}}\mathrm{CO}2 $$ ratio
GOLD: Global Initiative for Chronic Lung Disease
FVC: Forced vital capacity
CPET: Cardiopulmonary exercise test
OCD: Oxygen-cost diagram
TLC: Total lung capacity
RV: Residual volume
D_L_CO: Diffusing capacity for carbon monoxide
P_ET_CO_2_: End-tidal PCO_2_
S_P_O_2_: Arterial oxyhemoglobin saturation was measured by pulse oximetry
RCP or VCP: Respiratory or ventilatory compensation point
$$ \dot{\mathrm{V}}\mathrm{E}/\dot{\mathrm{V}}\mathrm{CO}2\mathrm{N} $$: $$ \dot{\mathrm{V}}\mathrm{E}/\dot{\mathrm{V}}\mathrm{CO}2 $$ nadir
