# Supplementary material for: Mechanisms affecting exercise ventilatory inefficiency-airflow obstruction relationship in male patients with chronic obstructive pulmonary disease
Source: Respir Res. 2020 Aug 6;21:206. doi: 10.1186/s12931-020-01463-4 (PMC7409645; doi:10.1186/s12931-020-01463-4)
Supplement: Supplementary file 1 — Additional file 1: Supplementary Table. Summary of correlation (r) of $\dot{\mathrm{V}}\mathrm{E}/\dot{\mathrm{V}}\mathrm{CO}2$V˙E/V˙CO2 slope ($\dot{\mathrm{V}}\mathrm{E}/\dot{\mathrm{V}}\mathrm{CO}2\mathrm{S}$V˙E/V˙CO2S) and its intercept ($\dot{\mathrm{V}}\mathrm{E}/\dot{\mathrm{V}}\mathrm{CO}2\mathrm{I}$V˙E/V˙CO2I) with pulmonary physiology. [file 12931_2020_1463_MOESM1_ESM.docx]

Supplementary Table. Summary of correlation (r) ofslope () and its intercept () with pulmonary physiology.

| r | | Slope | | | | | | | | |  | Intercept | | |
| --- | --- | --- | --- | --- | --- | --- | --- | --- | --- | --- | --- | --- | --- | --- |
| N = | | 316[^11^](#_ENREF_11) | 52[^13^](#_ENREF_13) | | | | | | 46[^3^](#_ENREF_3) | 16[^12^](#_ENREF_12) |  | 316[^11^](#_ENREF_11) | 52[^13^](#_ENREF_13) | 46[^3^](#_ENREF_3) |
| Intercept | | -0.74^**^ | −0.25^¶^ | | | | | | -- | -- |  | 1 | 1 | -- |
| Expiration | FEV_1_% | NS^♣^ | NS | | | | | | -- | -- |  | -- | −0.48^***^ | -- |
| FEV_1_/VC | | -- | -- | | | | | | NS | P |  | -- | -- | −0.38^**^ |
| GOLD | | ^♦^ | -- | | | | | | -- | -- |  | -- | -- | -- |
| Inspiration | V_T_/T_Ipeak_ | -- | -- | | | | | | -- | -- |  | -- | -- | -- |
| Volume excursion/dynamic hyperinflation: | | | | | |  | |  |  |  |  |  |  |  |
| V_Tpeak_/FEV_1_ | | -- | −0.33^*^ | | | | | | -- | N |  | -- | 0.43^***^ | -- |
| V_Tpeak_/IC | | -- | −0.35^*^ | | | | | | -- |  |  | -- | NS | -- |
| V_Tpeak_/VC | | -- | −0.31^*^ | | | | | | -- |  |  | -- | NS | -- |
| EELV_rest_/TLC | | -- | -- | | | | | | -- |  |  | -- | 0.34^*^ | -- |
| EELV_peak_/TLC^⇑^ | | −0.48-−0.60^*♣♣^ | -- | | | | | | -- |  |  | 0.62-0.75^**♣♣^ | 0.45^***^ | -- |
| Gas exchange: | | | | |  | |  | |  |  |  |  |  |  |
| S_P_O_2peak_^⇑^ | | −0.48^*^−0.60^*♣♣^ | -- | | | | | | -- |  |  | 0.62-0.75^**♣♣^ | -- | -- |
| P_ET_CO_2peak−rest_ | |  | −0.64^***^ | | | | | | -- | N |  |  | 0.59^***^ | -- |
| V_D_/V_Trest_ | | -- | -- | | | | | | -- | -- |  | -- | -- | -- |
| V_D_/V_Tpeak_ | | -- | -- | | | | | | -- | NS |  | -- | -- | -- |
| Exercise capacity | | | | |  | |  | |  |  |  |  |  |  |
| _peak_ | | −0.38^*^-−0.45^*♣♣^ | | −0.32^*^ | | | | | −0.34^*^ | -- |  | −0.58-−0.72^**♣♣^ | NS | NS |
| Work_peak_ | | -- | | −0.43^**^ | | | | | −0.51^***^ | -- |  | -- | NS | NS |

Abbreviations: please see Table 2. Slope calculation: in reference[^13^](#_ENREF_13), the linear regression was used for the data not mentioning the data of the entire loaded test or below the respiratory compensatory point (RCP). In reference[^3^](#_ENREF_3)^,^[^12^](#_ENREF_12), the data used for linear regression was below RCP. In reference[^11^](#_ENREF_11),versus FEV_1_%, r = 0.18, p > 0.05 in GOLD I only (N=81)[^11^](#_ENREF_11), not available for the other stages. ^♦^Correlation coefficient not formally reported in reference[^11^](#_ENREF_11) but highly possible to be significant in the reported figures. ^⊗^related to FEV_1_/VC, r = −0.377, p = 0.009.[^3^](#_ENREF_3) ^⇑^In reference[^11^](#_ENREF_11), corrected by V’_E peak_ for EELV_peak_/TLC, S_P_O_2peak−rest_ used in reference[^11^](#_ENREF_11), --: not available, ^♣^GOLD stage 1 and ^♣♣^GOLD stage 2-4 in reference[^11^](#_ENREF_11), P and N: In reference[^12^](#_ENREF_12), % of emphysema evaluated by HRCT were negatively related to FEV_1_/FVC and(r = -0.72 and -0.77) and positively related to P_ET_CO_2peak_ and V_T_/FEV_1_ (r = 0.98 and 0.74), and thus deduced the positive relationship (P) between FEV_1_/FVC andand negative relationship (N) between P_ET_CO_2peak_ andand between V_T_/FEV_1_ and. ^¶^p < 0.1 >0.05, ^*^< 0.05, ^**^< 0.01, ^***^≤0.001, ^🕆^< 0.0001.
