# Supplementary material for: Mechanisms affecting exercise ventilatory inefficiency-airflow obstruction relationship in male patients with chronic obstructive pulmonary disease
Source: Respir Res. 2020 Aug 6;21:206. doi: 10.1186/s12931-020-01463-4 (PMC7409645; doi:10.1186/s12931-020-01463-4)
Supplement: Supplementary file 2 — Additional file 2. [file 12931_2020_1463_MOESM2_ESM.pdf]

ID	Vd_VtMX	VD_VTmx_	O2Ppk_rs	Brg_VO2n	VT_TIMAX_	AGE	HT	WT
1001	0.546	0.04	3.434	12.74788	1.56515581	61	160	62.0
1002	0.609	-0.15	0.558	9.615385	1.91538462	65	157	41.0
1003	0.487	-0.17	3.194	11.30653	1.54899497	66	165	48.0
1004	0.522	-0.13	3.162	8.87199	1.61850444	72	163	48.0
1005	0.517	-0.05	3.688	9.782609	1.19565217	64	165	65.0
1006	0.531	-0.09	5.61	10.33058	1.34504132	67	160	56.0
1007	0.407	-0.15	5.395	9.584665	1.96911608	67	176	72.0
1008	0.477	-0.07	3.35	5.741627	1.59808612	64	166	67.0
1009	0.608	-0.03	1.775	18.82353	1.35764706	68	164	54.0
1010	0.493	-0.11	5.062	10.6383	1.71702128	67	169	61.0
1011	0.490	0.01	3.397	12.13592	1.53398058	51	164	49.0
1012	0.648	-0.03	2.093	19.01141	1.58745247	63	172	58
1013	0.491	-0.16	5.036	12.58581	1.64187643	73	172	63
1014	0.447	-0.13	3.687	10.43841	1.77870564	67	156	55
1015	0.451	-0.16	3.274	10.36269	1.4984456	70	174	61
1016	0.481	-0.07	4.134	12.44344	1.46945701	67	160	60
1017		0.00	7.037	8.902077	1.71018793	50	168	55
1018	0.500	-0.07	4.22	12.57862	1.25408805	67	161	55
1019	0.569	0.11	1.928	12.56732	2.1561939	55	175	69
1020	0.424	-0.02	3.031	12.85714	1.19857143	63	168	59
1021	0.363	-0.04	3.352	6.216006	1.68376068	62	176	60
1022	0.586	0.03	2.099	15.29637	1.71892925	63	168	54
1023	0.462	-0.16	5.425	10.28037	1.99906542	71	162	63
1024	0.405	-0.28	5.009	8.38223	1.34534786	67	166	55.0
1025	0.331	-0.29	7.75	4.36409	1.36346633	62	160	58.0
1026	0.336	-0.29	8.854	5.442177	1.29591837	69	162	67.5
1027	0.363	-0.18	7.364	5.8862	1.4911707	76	166	65.0
1028	0.468	-0.11	4.971	5.529226	1.32701422	64	160	52.0
1029	0.220	-0.25	5.255	8.211679	1.01277372	67	160	56.0
1030	0.458	-0.20	3.424	11.72333	2.12778429	69	161	44.0
1031	0.252	-0.23	5.978	5.904059	0.97638376	64	165	53.0
1032	0.389	-0.25	6.761	5.098325	1.40932265	67	165	50.0
1033	0.307	-0.19	7.976	4.393191	1.41625481	69	173	65.0
1034	0.347	-0.26	5.053	8.347245	1.16777963	62	166	66.0
1035		-0.53	9.454	5.063291	1.18177215	52	171	75.0
1036	0.462	-0.04	5.899	6.092917	1.2033511	75	173	70.0
1037	0.443	-0.17	5.917	8.445946	1.55236486	68	162	46
1038	0.456	-0.15	5.555	9.478673	1.30995261	63	161	65
1039	0.467	-0.26	4.972	9.060023	0.99207248	66	150	59
1040	0.289	-0.31	3.528	3.386005	1.14108352	75	147	41
1041	0.417	-0.24	6.716	3.053435	1.29923664	65	164	87
1042	0.367	-0.25	6.085	3.109453	1.34950249	60	174	92
1043	0.188	-0.31	6.015	4.250152	0.96964177	57	173	86

1044	**0.360**	-0.22	9.185	7.372654	1.21648794	61	167	54
1045	**0.369**	-0.23	4.721	9.066183	1.67089755	66	160	59
1046	**0.345**	-0.15	8.674	9.632224	1.25744308	71	164	78

BMI	SMOKE	XIMUM po	OCD	TRICEP	MIDARM	BORGRS	BORGLT	BORGMX
24.22	45	0	3	5.50	28.00	0	0.0	9
16.63	15	2	6	2.50	21.50	0	2.0	5
17.63	50	2	8	3.20	25.00	0	1.0	9
18.07	50	4	7	4.50	24.00	0	0.0	7
23.88	40	2	7	7.50	29.00	0	1.0	9
21.88	45	2	7	6.00	18.00	0	3.0	10
23.24	40	4	8	8.50	31.00	0	0.0	9
24.31	30	3	9	8.00	31.00	0	3.0	6
20.08	40	0	6	8.00	26.00	0	1.5	8
21.36	50	2	9	6.00	28.00	0	8.0	10
18.22	33	1	8	3.20	23.00	0	2.0	10
19.61	80	1	6	3.5	28	0	3.0	10
21.30	56	2	6	4	30	0	1.0	11
22.60	40	7	8	7	25.5	0	0.0	10
20.15	50	4	8	6	24.5	0	3.0	10
23.44	50	4	6	5.5	26.5	0.5	1.0	11
19.49	20	1	7			0	5	9
21.22	40	1	5.5	6.5	27.5	0	7	10
22.53	10	0	5	5.5	28.5	0.5		7
20.90	40	1	5	4	26	0	3	9
19.37	40	6	7			0	2	8
19.13	40	1	7	7.5	26.5	0	0.5	8
24.01	114	1	8	8	28.5	0	2	11
19.96	30	5	7	4.00	28.00	0	1.0	10
22.66	25	4	8	3.50	28.00	0	4.0	7
25.72	45	0	6	7.50	28.50	0	2.0	8
23.59	60	1	5	4.10	28.00	1	6.0	9
20.31	47	2	6	2.50	25.50	0	4.5	7
21.88	19	2	7	4.50	25.00	0	3.5	9
16.97	40	5	6	2.50	24.00	0	1.5	10
19.47	15	3	7.5	7.00	27.00	0	2.5	8
18.37	50	5	10	5.50	26.00	0	1.0	7
21.72	19	6	9	5.50	29.00	0	2.0	8
23.95	40	2	8	8.00	31.00	0	3.0	10
25.65	30	4	9	8.50	18.00	0	3.0	10
23.39	60	0	9	11.0	30.00	0	1.0	8
17.53	40	3	7	3.5	25	0	9.0	10
25.08	20	1	8.5	10.5	30.5	0	4.0	10
26.22	75	1	7.3	9	32	0	1	8
18.97	40	5	5	4.5	25.5	0	3	3
32.35	40	4	8.5	12	32	0	4	4
30.39	80	7	6	11.5	33.5	1	2	5
28.73	15	5	6	7	32	0	3	7

19.36	45	4	8	6.5	25.5	0	1	11
23.05	41	7	7	9	28.5	0	0	10
29.00	51	0	7	12	32.5	0	0.5	11

BORG3	BORG7	HTR_36_	CTR__	DPA_1.8c	_15 cm2	_10cm2	_13 cm2	_8 cm2
1	0	0.3803	0.4328	1.4	18.5	8.68	13.7	8.89
0.5	0.5	0.4235	0.4392	1.4	10.7	6.35	9.81	6.23
2	0	0.3956	0.4249	2	15.2	10	17.4	6.89
1	0	0.3581	0.5152	2.1	17.1	8.06	13.2	8.95
1	0	0.3608	0.3436	1.4	6.49	3.65	11.4	5.83
3	0	0.3422	0.4551	1.5	12.3	7.9	15.2	6.67
0	0	0.376	0.522	1.5	17.6	7.9	17.9	10.5
0.5	0	0.3547	0.4122	1.4	12.2	8	11.6	8.54
5	1	0.3548	0.5018	1.8	11.9	7.29	12.5	9.21
4	1	0.3737	0.4184	1.4	12.8	7.9	12.6	7.54
0	0	0.3207	0.3517	1.6	13.5	8.65	15.6	8.28
3	0	0.374	0.351	1.3	12.2	7.39	12.7	7.76
3	0	0.43	0.35	1.5	10.2	7.88	13.5	5.98
1	0	0.3692	0.448	1.4	11.1	6.75	10.3	7.29
2	0	0.3168	0.429	1.4	10.7	7.12	12.9	6.38
6	0.5	0.3576	0.5208	1.8	8.83	4.49	15.3	6.09
		0.361	0.358	1.4	10.1	5.4	8.7	5.4
6	0	0.283	0.424	2	11.3	9.45	13.2	9.65
3	2	0.344	0.491	2				
2	0			1	10.8	7.82	11.5	6.55
2	0	0.427		1.2				
8	1	0.301	0.425	1.4	16.9	8.89	13.5	8.64
3	0		0.478	1.7	9.12	5.64	10.6	7.17
2	0	0.3698	0.4204	1.5	10.6	5.24	14.3	5.47
0.5	1	0.383		2.5	19.8	7.51	19.7	11.7
1	1	0.34	0.4548	1.55	15.2	5.83	20.3	8.25
1	1	0.3729	0.4814	1.8	26.2	12	21.7	8.97
2	0	0.3922	0.422	1.65	13.2	7.87	14.8	8.64
2	0.5	0.3621	0.4086	1.7	16.9	10.8	17.3	9.64
0.5	0	0.4297	0.3954	1.6	10.2	5.76	10.1	4.73
1	0	0.3523	0.3617	1.3	9.13	5.08	12.8	5.36
2	0	0.3682	0.4404	1.4	12.2	9.7	10.2	4.57
2	0	0.2823		1.8	14.4	7.19	12.8	4.7
3	0	0.374	0.514	1.3	11.4	5.76	14.3	5.92
0.5	0	0.3312	0.4841	1.4	17.5	10.5	17	8.15
1	0	0.3541	0.4295	1.6	14.2	9.87	14.2	8.11
4	1	0.3913	0.36956	2	13.7	9.67	13.9	6.4
1.5	0	0.33	0.446	1.7	11.4	9.35	24	14.4
2	0	0.394	0.56	2.5	17.4	9.98	17.4	12.3
1	1	0.367	0.367	1.6				
2	1	0.407	0.607	2.5				
0.5	0	0.385		1.6	16.9	8.36	17.1	6.76
2	0	0.379	0.548	1.5	12.5	7.04	13.5	9.19

5	1	0.279	0.364	1.4	10.9	7.29	9.48	6.63
		0.351	0.466	1.4	16.3	9.67	13.4	4.82
2	0	0.338	0.495	1.5	17.9	9.6	13.3	6.9

IVS	_6 mm	TR	TLC	TLCpred	FRC	FRCpred	IC	RV
0	3.45	0	6.62	1.33	5.29	1.81	1.33	4.04
0	6.74	1	4.48	1.14	3.21	1.18	1.27	2.53
0	4	1	8.8	1.63	7.72	2.16	1.08	6.2
1	7.28	1	5.49	1.09	3.26	0.97	2.23	3.1
0	7.31	0	7.18	1.56	6.15	2.26	1.03	4.55
0	6.1	1	6.54	1.58	4.5	1.76	2.04	3.47
0	6.9	1	6.86	1.28	4.4	1.39	2.46	3.37
0	7.14	0	6.52	1.36	4.32	1.52	2.2	3.64
0	7.27	1	6.97	1.53	5.5	1.88	1.47	3.98
0	5.37	1	7.1	1.43	4.72	1.53	2.38	4.4
0	10	1	7.25	1.52	5.26	1.74	1.99	4.44
0	5.14	1	5.75	1.1	4.72	1.44	1.03	3.7
0	5	0	8.1	1.59	6.12	1.91	1.98	5.52
0	8.3	1	5.08	1.36	3.65	1.58	1.43	2.86
0	6.4	0	8.48	1.8	6.72	2.26	1.76	5.39
0	9.6	1	4.97	1.07	3.97	1.43	1	3.44
0	4.9	1	6.87	1.38	5.57	1.83	1.3	3.26
0	4.5	1	6	1.24	5.04	1.68	0.96	4.17
			8.18	1.482	6.69	2.07	1.49	5.64
0	8	1	7.09	1.604	6.07	2.2	1.02	4.91
			7.76	1.429	5.21	1.53	2.55	3.89
0	2.74	1	6.81	1.42	5.88	1.95	0.93	5.44
0	5.94	1	6.51	1.51	4.73	1.81	1.78	4.41
	7	0	6.87	1.29	5.09	1.49	1.78	4.27
0	3.11	1	6.5	1.55	4.88	1.89	1.62	3.27
0	6.5	1	6.36	1.31	4.37	1.57	1.99	3.8
0	3.9	1	7.05	1.24	4.63	1.3	2.42	3.06
0	6	1	5.74	1.37	4.3	1.39	1.44	3.59
0	5	1	7.06	1.45	5.57	1.83	1.49	4.39
0	6	1	5.65	1.37	4.52	1.63	1.13	2.77
0	2.95	0	6.6	1.22	4.92	1.42	1.68	3.82
0	3	1	6.23	1.22	3.36	1.02	2.87	2.79
0	3.32	0	8.71	1.5	6.01	1.63	2.7	3.08
0	6	0	5.29	1.15	4.36	1.63	0.93	3.29
0	6.88	0	6.05	1.17	4.11	1.48	1.94	3.05
0	10	1	6.01	1.24	4.5	1.53	1.51	4.28
0	6.8	1	6.02	1.33	4.14	1.35	1.88	2.87
1	7.5	0	5.69	1.05	3.99	1.24	1.7	3.57
0	1.3	1	5.07	1.3	3.63	1.57	1.44	2.78
			5.51	1.7	4.43	2.13	1.08	3.08
		1	5.38	0.998	3.69	1.3	1.69	2.78
	4	1	6.23	0.972	3.78	1.12	2.45	3.12
0	6.86	0	6.59	0.918	3.59	0.9	3	2.99

0	5.9	0	8.31	1.68	6.35	2.07	1.96	5.62
0	7.77	0	6.06	1.38	4.38	1.62	1.68	2.78
0	9	1	5.54	1.23	4.07	1.62	1.47	3.16

RV_TLC	DLCO	DLCOpred	DCOprct_(	FVC	FVCpred	FEV1	FEV1pred	FEV1_
0.61	9.6	0.42	0.41	2.58	0.81	0.88	0.36	0.34
0.56	15.8	0.75	0.71	1.95	0.81	1.04	0.56	0.53
0.7	8.8	0.37	0.38	2.6	0.77	0.88	0.34	0.34
0.56	10.2	0.5	0.47	2.3	0.76	1.3	0.56	0.57
0.63	19.4	0.8	0.82	2.39	0.82	0.73	0.32	0.31
0.53	20.2	0.93	0.9	2.69	1.07	1.26	0.65	0.47
0.49	13.4	0.5	0.54	3.49	1.03	1.81	0.69	0.52
0.56	17.9	0.71	0.75	2.84	0.93	1.74	0.74	0.61
0.57	8.1	0.35	0.36	2.5	0.89	0.92	0.43	0.37
0.62	13.7	0.54	0.58	2.35	0.76	1.34	0.56	0.57
0.61	13.3	0.5	0.51	2.81	0.87	1.19	0.47	0.42
0.64	11.3	0.42	0.45	1.46	0.44	0.58	0.22	0.4
0.68	19.8	0.79	0.86	1.98	0.64	0.95	0.4	0.48
0.56				2.19	1	1.47	0.85	0.67
0.64	16.3	0.68	0.68	3.09	1.07	0.99	0.44	0.32
0.69	15.6	0.71	0.7	1.57	0.48	0.75	0.32	0.48
0.47	20.4	0.73	0.75	3.83	1.15	2.5	0.886	0.73
0.695	12.4	0.82	0.55	2.08	0.644	0.62	0.253	0.3
0.69	5.3	0.24	0.19	2.69	0.68	0.54	0.171	0.2
0.69	13.6	0.57	0.56	1.58	0.44	0.8	0.288	0.77
0.5	23.4	0.84	0.9	3.59	1.02	2.74	1	0.8
0.8	7.9	0.31	0.32	2.47	0.81	0.73	0.31	0.3
0.68	12.4	0.79	0.57	1.58	0.61	0.86	0.43	0.54
0.62	15.9	0.68	0.68	2.5	0.76	1.14	0.45	0.46
0.5	22.4	0.99	0.96	3.02	1.14	1.42	0.69	0.47
0.6	18.7	0.87	0.84	1.99	0.62	0.88	0.37	0.44
0.43	20.5	0.87	0.95	3.84	1.13	1.86	0.72	0.48
0.63	17.8	0.79	0.78	2.1	0.68	0.92	0.39	0.44
0.62	12	0.55	0.54	1.96	0.65	0.79	0.34	0.4
0.49	9.3	0.44	0.42	2.86	1.16	1.11	0.59	0.39
0.58	14.8	0.61	0.62	2.74	0.8	1.08	0.41	0.39
0.45	12.2	0.54	0.53	3.1	0.98	1.82	0.75	0.59
0.35	21.3	0.83	0.88	4.28	1.14	1.86	0.64	0.43
0.62	21.9	0.89	0.9	2	0.68	1.32	0.57	0.66
0.5	32.1	1.14	1.18	3	0.86	1.66	0.6	0.55
0.71	15.7	0.66	0.69	1.63	0.56	1.05	0.47	0.64
0.48	11.4	0.49	0.51	3.02	1.08	1.61	0.75	0.53
0.63	19.9	1.1	0.85	1.85	0.52	0.64	0.22	0.35
0.55	14.6	1.02	0.7	2.11	0.92	1.04	0.59	0.49
0.56	6.4	0.66	0.34	2.15	0.96	1.13	0.576	0.53
0.52	20.3	0.85	0.87	1.76	0.52	1.16	0.448	0.66
0.5	17.2	0.61	0.66	2.05	0.53	0.84	0.279	0.41
0.45	28.7	0.91	1.08	3.45	0.89	1.49	0.49	0.43

0.69	12.9	0.5	0.52	2.22	0.71	1.19	0.49	0.54
0.46	15.1	0.65	0.67	2.24	0.82	1.07	0.51	0.48
0.57	19.2	1	0.87	1.9	0.7	1.1	0.53	0.58

SVC	SVCpred	MIPcmH2(	MEPcmH2	POSTMIP	POSTMEF	PEFR	PFRpred	LA_RSWI
2.58	0.81	62	148	53	132	2.6	0.38	0.57
1.95	0.81	50	70	35	63	2.87	0.52	
2.6	0.77	70	93	70	101	1.66	0.23	0.68
2.3	0.76	48	71			2.54	0.38	0.45
2.63	0.91	55	94	60	106	1.4	0.17	0.36
3.07	1.22	46	93			1.59	0.21	0.29
3.49	1.03	86	105	80	110	4.15	0.46	0.39
2.88	0.95	99	104	90	106	2.25	0.27	0.35
2.99	1.07	39	80	55	91	3.35	0.55	0.37
2.7	0.87	91	122	83	128	3.12	0.38	0.38
2.81	0.87	75	83	79	78	3.02	0.36	0.37
2.05	0.61	54	121	71	129	1.18	0.27	
2.58	0.83	60	94	70	119	1.61	0.25	0.37
2.22	1	80	92	81	82	4.13	0.78	0.19
3.09	1.07	66	81	75	118	2.34	0.38	0.5
1.5	0.54	52	129	87	104	1.15	0.15	0.43
3.61	1.07	42	108	39	108	4.55	0.533	0.31
1.83	0.62	50	92	61	98	1.71	0.221	0.23
2.54	0.46					1.11	0.126	0.37
2.18	0.776	42	56	88	74	1.5	0.182	0.24
3.87	1.102	61	84	44	81	7.3	1.034	
1.37	0.45	45	126	47	128	2.19	0.34	0.57
2.1	0.81	108	160	115	214	2.92	0.51	0.6
2.6	0.79	71	99	58	109	2.45	0.31	0.46
3.23	1.22	72	69	88	75	4.03	0.69	0.3
2.56	0.87	49	125	97	129	3.05	0.4	0.51
3.99	1.18	68	75	81	92	3.19	0.43	0.95
2.15	0.7	84	88			2.5	0.37	0.4
2.67	0.89	87	111	81	84	1.58	0.2	0.43
2.88	1.17	59	97	52	99	1.57	0.2	0.64
2.78	0.82	51	107	71	101	2.09	0.26	0.52
3.44	1.09	74	104			4.65	0.59	0.34
4.6	1.22	82	114	93	136	4.28	0.55	0.46
2	0.68	84	133	72	131	4.6	0.56	0.35
3	0.86	86	130	102	109	5.78	0.83	0.46
1.73	0.59	76	117	106	121	3.17	0.39	0.52
3.15	1.13	85	105	91	81	3.94	0.65	0.25
2.12	0.62	91	145			1.68	0.2	0.22
2.29	0.99	55	80	68	91	2.3	0.65	0.21
2.42	1.36			42	58	2.99	0.72	0.54
2.6	0.769	57	119	62	113	4.08	0.563	0.4
3.11	0.748	87	117	74	110	2.64	0.305	0.44
3.6	0.755	108	111	112	89	3.75	0.408	0.69

2.51	0.81	84	101	69	102	4.01	0.62	0.33
3.28	1.2	52	108	56	97	2.35	0.41	0.38
2.38	0.87					1.88	0.31	0.46

LACLTW	LACMXW	LARSPL	LACLTPL	LACMXP	LAMX_R	PH_REST	PH_LT	PH_MAX
	1.9	1.28		3.61	2.33	7.4		7.354
		0.97		4.37	3.4	7.38		7.316
0.9	3.35	1.64	1.87	6.42	4.78	7.36	7.357	7.274
0.85	3.35	1.1	1.75	5.55	4.45	7.35	7.337	7.282
0.57	2.58	1.27	1.4	5.57	4.3	7.37	7.356	7.285
0.35	1.76	0.7	0.83	2.97	2.27	7.36	7.351	7.277
0.68	3.01	1.16	1.39	5.2	4.04	7.41	7.414	7.361
0.46	2.83	0.92	1.17	4.82	3.9	7.41	7.389	7.315
0.32	1.11	1.01	1.06	2.66	1.65	7.42	7.411	7.377
0.68	2.32	0.9	1.2	4.24	3.34	7.39	7.379	7.346
0.79	2.87	1.06	1.61	4.76	3.7	7.43	7.402	7.351
						7.38		7.334
0.83	2.11	1.63	1.75	4.51	2.88	7.39	7.391	7.334
0.41	5.38	1.34	1.44	8.04	6.7	7.41	7.44	7.34
0.65	3.05	1.59	1.76	5.75	4.16	7.41	7.406	7.319
0.36	3.11	1.12	1.07	5.91	4.79	7.45	7.423	7.328
0.5	1.68	0.9	1.06	3.78	2.88			
0.79	2.3	0.93	1.77	4.66	3.73	7.39	7.357	7.287
0.39	0.97	1.02	0.75	1.49	0.47	7.46	7.398	7.384
0.36	0.91	0.72	1.05	1.99	1.27	7.39	7.382	7.336
		1.65	3.79	9.81	8.16	7.42	7.392	7.319
0.57	1.9	1.18	1.16	3.24	2.06	7.38	7.382	7.325
0.63	2.52	1.59	1.32	4.41	2.82	7.43	7.406	7.364
0.87	3.61	1.01	1.48	6.11	5.1	7.41	7.368	7.27
0.47	3.18			5.6		7.44	7.401	7.318
0.58	1.8	1.96	1.7	3.93	1.97	7.41	7.421	7.376
	2.99	1.88		4.64	2.76	7.4		7.352
0.44	3.25	0.95	1.09	5.93	4.98	7.35	7.34	7.25
0.78	2.56	1.04	1.43	4.08	3.04	7.4	7.375	7.306
1.06	4.06	1.17	2.09	6.25	5.08	7.39	7.376	7.305
0.95	3.52					7.37	7.349	7.287
0.65	4.13	1.17	1.44	7.96	6.79	7.42	7.399	7.329
0.74	4.67	0.83	1.57	7.4	6.57	7.43	7.388	7.265
0.6	2.48	0.86	1.19	4.69	3.83	7.39	7.377	7.338
0.65	2.51	1.26	1.31	4.18	2.92	7.43	7.414	
	1.71	1.04		2.72	1.68	7.38		7.36
0.55	3.07	1.36	1.39	5.53	4.17	7.39	7.386	7.312
0.66	3.14	0.73	1.24	4.49	3.76	7.38	7.348	7.292
0.3	0.7	0.98	1.03	1.87	0.89	7.3	7.301	7.275
	3.61	1.47		6.63	5.16	7.39		7.284
	3.18	1.634		6.99	5.36	7.37		7.326
1.26	4.87	1.13	2.56	8.25	7.12	7.38	7.356	7.252
0.77	3.8	1.5	1.77	5.66	4.16	7.37	7.353	7.261

0.61	3.56	0.84	1.21	6.28	5.44	7.39	7.382	7.294
0.69	5.4	1.22	1.67	10.7	9.5	7.42	7.4	7.322
0.41	2.18	1.07	1.07	3.96	2.89	7.4	7.409	7.353

PHMX_RS	PaCO2RS	PaCO2LT	PaCO2MX	PCO2mx_	PaO2RST	PaO2LT	PaO2_MX	PO2rst_m
0.046	40		53	13	57		45	13
0.062	41		44	4	82		64	18
0.085	44	47	53	9	74	71	58	15
0.066	42	44	47	5	84	87	83	1
0.08	51	50	60	9	87	82	56	31
0.086	39	39	49	10	92	87	59	32
0.052	33	33	32	-1	84	76	68	16
0.098	33	39	43	10	89	81	77	13
0.039	43	43	48	5	71	68	56	15
0.043	39	38	39	0	85	89	82	2
0.082	39	42	47	7	82	77	71	11
0.043	54		61	7	67		53	15
0.054	39	36	41	2	75	74	76	-2
0.07	37	35	37	0	91	91	98	-7
0.092	36	35	40	4	84	84	90	-6
0.126	40	37	53	13	75	74	65	10
0.104	46	49	57	11	80	70	57	23
0.077	28	38	41	12	73	69	60	13
0.05	33	42	43	11	88	79	64	24
0.102	30	37	34	5	99	87	78	22
0.056	43	42	49	6	83	79	79	4
0.065	37	38	41	4	80	73	72	7
0.136	42	44	49	7	90	83	62	28
0.123	33	39	39	5	94	105	94	0
0.038	39	37	40	1	68	71	76	-8
0.05	39		39	1	80		88	
0.102	46	46	55	9	76	76	75	1
0.094	33	36	40	7	74	68	64	10
0.085	41	38	39	-2	84	90	74	10
0.085	43	43	47	4	78	74	56	21
0.089	40	40	42	2	83	89	70	13
0.165	35	40	44	9	95	78	65	30
0.05	42	44	42	0	83	85	84	-1
	34	34			62	71		
0.015	43		49	6	92		102	-10
0.081	39	34	44	6	81	81	57	25
0.09	48	52	57	9	74	75	69	5
0.027	64	63	65	2	51	50	42	10
0.106	36		32	-4	79		75	4
0.048	48		60	12	66		58	7
0.125	44	44	50	6	73	66	57	15
0.111	47	47	54	6	67	63	53	13

0.099	41	45	45	4	87	81	66	21
0.097	41	45	40	-1	81	79	88	-7
0.044	44	43	43	-1	70	69	84	-15

HCO3RS	HCO3LT	HCO3MX	BICBMX_	SaO2RS	SaO2LT	SaO2_MX	SO2rst_m›	SBPRS
24		26	-2	90		78	12	134
24		22	2	96		91	5	127
24	25	22	2	94	94	86	8	143
23	23	21	2	96	96	95	1	134
27	26	25	3	96	96	85	11	142
23	22	21	2	97	96	87	10	130
23	23	20	3	97	95	93	3	156
23	24	21	2	97	96	94	3	150
27	27	27	1	95	94	88	6	158
24	23	22	2	96	97	96	1	152
27	26	25	2	96	95	95	1	123
29		28	1	93		84	9	125
24	23	21	3	95	95	95	0	167
25	25	20	4	97	97	97	0	125
24	23	20	4	96	96	96	0	140
28	25	25	3	96	95	91	5	145
								131
27	26	24	3	96	93	86	10	123
23	24	24	-1	96	94	91	5	134
21	25	22	-1	99	96	91	7	166
22	23	19	3	98	97	95	3	120
25	25	24	1	96	95	95	1	147
26	24	23	3	96	95	94	2	174
26	24	20	6	97	96	89	8	135
25	24	20	5	97	98	99	-1	128
25	25	23	2	94	95	95	-1	161
25		22	3	96		96	-1	150
24	24	21	3	95	94	92	2	120
22	22	20	2	95	93	90	5	126
25	23	19	5	96	97	94	3	125
25	23	21	4	95	94	86	9	159
26	25	21	5	96	97	93	4	178
24	24	19	6	97	95	90	7	132
25	25	22	3	96	96	96	0	145
24	23	22	2	92	95			148
25		26	-1	97		97	-1	160
24	22	21	3	96	96	87	9	133
27	26	24	3	98	94	92	7	157
27	27	25	2	82	80	69	12	161
23		18	5	96		98	-3	204
26		28	-1	92		88	5	158
21	24	20	1	94	92	85	9	156
21	25	21	0	93	91	82	10	178

25	26	21	4	97	96	91	6	128
27	27	21	6	96	96	96	0	125
27	27	23	3	94	94	96	-2	156

SBPLT	SBP_MA	DBP_RST	DBPLT	DBP_MA	HR_RST	HRRS_M	HRLT	HRLT_M
	183	88		106	102	0.6		
	164	80		94	75	0.449		
166	232	78	83	109	88	0.527	99	0.593
185	268	63	77	100	87	0.534	98	0.601
165	229	77	87	101	92	0.548	108	0.643
153	212	74	81	114	70	0.422	76	0.458
178	239	99	95	105	96	0.578	100	0.602
178	252	85	88	104	70	0.417	85	0.506
176	240	88	95	127	99	0.586	102	0.604
174	219	80	81	91	73	0.44	83	0.5
145	150	78	81	96	63	0.356	85	0.48
	217	88		110	78	0.497		
207	244	75	78	84	60	0.408	77	0.524
130	184	59	63	77	65	0.425	75	0.49
209	249	94	100	123	90	0.6	95	0.633
169	274	76	82	114	80	0.523	92	0.601
145	153	90	85	82	85	0.5	89	0.524
156	205	74	84	104	78	0.47	89	0.536
164	166	95	98	94	101	0.58	103	0.592
201	260	88	104	120	60	0.353	81	0.476
153	178	80	85	87	99	0.566	100	0.571
163	225	83	90	111	82	0.485	86	0.509
189	244	69	80	97	85	0.57	97	0.651
191	288	71	79	94	83	0.5	104	0.627
170	210	71	85	108	61	0.359	77	0.453
195	231	90	102	109	70	0.476	79	0.537
	230	87		100	88	0.547		
145	211	67	67	95	65	0.417	71	0.455
168	203	71	79	96	85	0.512	98	0.59
145	225	63	64	95	76	0.461	87	0.527
196	234	79	89	101	81	0.482	113	0.673
186	238	87	89	114	65	0.394	79	0.479
184	252	76	87	109	83	0.503	100	0.606
172	201	80	84	85	83	0.488	101	0.594
172		80	83		76	0.432	94	0.534
	241	75		73	63	0.391		
137	200	79	77	99	68	0.447	78	0.513
188	237	75	85	105	89	0.527	106	0.627
178	197	85	97	98	67	0.435	95	0.617
	226	94		96	105	0.652		
	230	83		104	103	0.613		
203	257	70	98	121	88	0.515	109	0.637
219	252		109	120	99	0.572	105	0.607

155	197	69	69	77	66	0.388	82	0.482
137	163	83	84	96	96	0.623	117	0.76
176	215	74	83	85	69	0.463	76	0.51

HR_PK	HRPK_M:	HRPRDMX	VO2MXPRD	VO2_RST	VO2RS_MX	VO2ATVS
122	0.718	170	1630	240	0.147	400
158	0.946	167	1016	205	0.202	400
136	0.814	167	1349	234	0.173	470
133	0.816	163	1161	241	0.208	519
141	0.839	168	1718	261	0.152	
109	0.657	166	1353	229	0.169	720
121	0.729	166	2057	227	0.11	548
151	0.899	168	1781	250	0.14	855
112	0.663	169	1242	200	0.161	380
118	0.711	166	1523	212	0.139	711
123	0.695	177	1538	208	0.135	549
102	0.65	157	1806	239	0.132	411
102	0.694	147	1648	212	0.129	472
134	0.876	153	1234	225	0.182	586
153	1.02	150	1579	273	0.173	679
134	0.876	153	1406	197	0.14	488
111	0.653	170	1775	176	0.099	875
108	0.651	166	1209	245	0.203	651
124	0.713	174	2251	259	0.115	
104	0.612	170	1575	222	0.141	570
177	1.011	175	1980	388	0.196	759
108	0.639	169	1457	225	0.154	390
127	0.852	149	1444	255	0.177	680
157	0.946	166	1484	215	0.145	772
135	0.794	170	1633	252	0.154	887
115	0.782	147	1730	275	0.159	850
149	0.925	161	1467	255	0.174	1114
126	0.808	156	1346	330	0.245	950
130	0.783	166	1373	270	0.197	710
135	0.818	165	954	220	0.231	568
144	0.857	168	1340	278	0.207	838
142	0.861	165	1175	189	0.161	859
162	0.982	165	1930	271	0.14	1051
157	0.924	170	1958	214	0.109	599
152	0.864	176	2334	269	0.115	1165
121	0.752	161	1610	312	0.194	800
133	0.875	152	1190	203	0.171	909
136	0.805	169	1491	196	0.131	710
119	0.773	154	1183	164	0.139	
160	0.994	161	936	211	0.225	838
138	0.821	168	1938	286	0.148	483
162	0.947	171	2180	338	0.155	1100
173	1	173	2480	347	0.14	933

122	0.718	170	1628	201	0.123	700
158	1.026	154	1583	217	0.137	608
92	0.617	149	1929	258	0.134	480

VSLP_MX	VO2LT	VO2LT_MX	VO2PEAK	VO2PK_M	VO2/KGRS
0.245			706	0.433	3.871
0.394			520	0.512	5
0.348	467	0.346	796	0.59	4.875
0.447	361	0.311	789	0.68	5.021
	369	0.215	920	0.536	4.015
0.532	428	0.316	968	0.715	4.089
0.266	479	0.233	939	0.456	3.153
0.48	385	0.216	1045	0.587	3.731
0.306	287	0.231	425	0.342	3.704
0.467	480	0.315	940	0.617	3.475
0.357	491	0.319	824	0.536	4.245
0.228			526	0.291	4.121
0.286	515	0.313	874	0.53	3.365
0.475	349	0.211	958	0.776	4.091
0.43	419	0.265	965	0.611	4.475
0.347	397	0.282	884	0.629	3.283
0.493	376	0.212	1011	0.57	3.259
0.538	446	0.369	795	0.658	4.455
	346	0.154	557	0.247	3.754
0.362	426	0.27	700	0.444	3.763
0.383	545	0.275	1287	0.65	6.467
0.268	230	0.158	523	0.359	4.167
0.471	475	0.329	1070	0.741	4.048
0.52	657	0.443	1193	0.804	3.909
0.543	638	0.391	1604	0.982	4.345
0.491	631	0.365	1470	0.85	4.074
0.759			1529	1.042	3.923
0.706	349	0.259	1266	0.941	6.346
0.517	575	0.419	1096	0.798	4.821
0.595	414	0.434	853	0.894	5
0.625	524	0.391	1355	1.011	5.245
0.731	393	0.334	1373	1.169	3.78
0.545	781	0.405	1821	0.944	4.169
0.306	578	0.295	1198	0.612	3.242
0.499	635	0.272	1975	0.846	3.587
0.497			1313	0.816	4.457
0.764	429	0.361	1184	0.995	4.413
0.476	609	0.408	1055	0.708	3.015
	441	0.373	883	0.746	2.78
0.895			886	0.947	5.146
0.249			1310	0.676	3.287
0.505	907	0.416	1608	0.738	3.674
0.376	507	0.204	1647	0.664	4.035

0.43	708	0.435	1492	0.916	3.722
0.384	575	0.363	1103	0.697	3.741
0.249	463	0.24	1142	0.592	3.308

VO2_KG_LT	VO2_KGM	VCO2_RST	VCO2LT	VCO2MA	RERRS	RERLT	RERMAX
	11.387	225		688	0.938		0.975
	12.683	152		510	0.741		0.981
9.729	16.583	196	410	867	0.838	0.878	1.089
7.521	16.438	200	364	909	0.83	1.008	1.152
5.677	14.154	240	332	864	0.92	0.9	0.939
7.643	17.286	189	342	888	0.825	0.799	0.917
6.653	13.042	175	429	1137	0.771	0.896	1.211
5.746	15.597	218	344	1158	0.872	0.894	1.108
5.315	7.87	179	246	396	0.895	0.857	0.932
7.869	15.41	176	464	1064	0.83	0.967	1.132
10.02	16.816	169	412	911	0.813	0.839	1.106
	9.069	194		456	0.81		0.86
8.175	13.873	173	483	910	0.82	0.938	1.04
4.727	17.418	174	298	1200	0.76	0.854	1.25
6.869	15.82	209	349	1040	0.77	0.833	1.08
6.617	14.733	176	362	980	0.89	0.912	1.11
6.963	18.722	145	369	1150	0.83	0.981	1.14
8.109	14.455	212	413	808	0.87	0.926	1.02
5.014	8.072	222	311	493	0.86	0.899	0.89
7.22	11.864	176	374	646	0.79	0.878	0.92
9.083	21.45	337	498	1553	0.87	0.914	1.21
4.259	9.685	196	205	497	0.88	0.891	0.95
7.54	16.984	225	378	1154	0.87	0.796	1.01
11.945	21.691	160	602	1315	0.744	0.916	1.102
11	27.655	200	539	1803	0.794	0.845	1.124
9.348	21.778	244	388	1400	0.887	0.615	0.952
	23.523	230		1548	0.902		1.012
6.712	24.346	281	310	1326	0.852	0.888	1.047
10.268	19.571	240	487	1063	0.889	0.847	0.97
9.409	19.386	187	397	1006	0.85	0.959	1.179
9.887	25.566	203	465	1423	0.73	0.887	1.05
7.86	27.46	147	370	1629	0.778	0.941	1.186
12.015	28.015	253	671	2190	0.934	0.859	1.203
8.758	18.152	172	486	1240	0.804	0.841	1.035
8.467	26.333	226	577	2031	0.84	0.909	1.028
	18.757	261		1268	0.837		0.966
9.326	25.739	159	370	1256	0.78	0.862	1.06
9.369	16.231	196	493	1078	0.78	0.81	1.02
7.475	14.966	126	372	815	0.77	0.844	0.92
	21.61	170		777	0.8		0.88
	15.057	213		1599	0.75		1.22
9.859	17.478	293	825	1965	0.87	0.91	1.22
5.895	19.151	292	456	1873	0.84	0.899	1.14

13.111	27.63	176	637	1532	0.87	0.9	1.03
9.914	19.017	169	454	1225	0.78	0.79	1.11
5.936	14.641	225	385	1154	0.87	0.832	1.01

WRLT	WRLT_M	WR_PK	WRPK_M	WRPRDM	O2PLSRS	O2PLSLT	O2PLSMX
		46	0.343	134	2.353		5.787
		40	0.417	96	2.733		3.291
0	0	71	0.573	124	2.659	4.717	5.853
11	0.101	75	0.686	109.4	2.77	3.684	5.932
0	0	85		143.3	2.837	3.417	6.525
0	0	94	0.701	134	3.271	5.632	8.881
26	0.149	92	0.526	175	2.365	4.79	7.76
0	0	133	0.899	148	3.571	4.529	6.921
0	0	17	0.137	124	2.02	2.814	3.795
36	0.247	96	0.658	146	2.904	5.783	7.966
43	0.295	88	0.603	146	3.302	5.776	6.699
		25	0.157	159	3.064		5.157
0	0	60	0.411	146	3.533	6.688	8.569
0	0	91	0.867	105	3.462	3.467	7.149
0	0	99	0.643	154	3.033	4.411	6.307
0	0	65	0.542	120	2.463	4.315	6.597
0	0	88	0.537	164	2.071	4.225	9.108
0	0	55	0.466	118	3.141	5.011	7.361
0	0	33	0.176	188	2.564	3.359	4.492
19	0.135	49	0.348	141	3.7	5.259	6.731
27	0.147	118	0.641	184	3.919	5.45	7.271
0	0	3	0.021	143	2.744	2.674	4.843
0	0	75	0.615	122	3	4.897	8.425
55	0.417	122	0.924	132	2.59	6.317	7.599
59	0.472	146	1.168	125	4.131	8.286	11.881
20	0.154	132	1.015	130	3.929	7.987	12.783
		128	1.008	127	2.898		10.262
6	0.051	105	0.89	118	5.077	4.915	10.048
28	0.239	81	0.692	117	3.176	5.867	8.431
30	0.286	72	0.686	105	2.895	4.759	6.319
39	0.295	107	0.811	132	3.432	4.637	9.41
23	0.19	128	1.058	121	2.908	4.975	9.669
65	0.419	198	1.277	155	3.265	7.81	11.241
40	0.267	124	0.827	150	2.578	5.723	7.631
43	0.229	185	0.984	188	3.539	6.755	12.993
		97	0.638	152	4.952		10.851
17	0.153	112	1.009	111	2.985	5.5	8.902
36	0.269	95	0.709	134	2.202	5.745	7.757
0	0	60	0.638	94	2.448	4.642	7.42
		70	1.273	55	2.01		5.538
		116	0.725	160	2.777		9.493
63	0.432	175	1.199	146	3.841	8.321	9.926
21	0.107	159	0.811	196	3.505	4.829	9.52

2	0.014	114	0.797	143	3.045	8.634	12.23
0	0	40	0.331	121	2.26	4.915	6.981
0	0	60	0.423	142	3.739	6.092	12.413

VO2_WR1L	VO2_WR2	VO2_WR_L	PaCO2RS	PaCO2LT	PaCO2MX	RERRS	RERLT
		5.901	39.8		52.7	0.938	
		3.682	40.5		44.1	0.741	
2.616	4.802	4.457	43.5	46.7	52.7	0.838	0.878
5.079	7.075	6.93	42.4	44.2	47	0.83	1.008
		5.94	50.6	50.2	59.8	0.92	0.9
5.3	6.245	6.145	39.4	38.7	48.9	0.825	0.799
5.45	7.409	6.929	32.8	33.1	32.2	0.771	0.896
6.409	4.347	6.641	33	39.4	43.3	0.872	0.894
		3.386	43.2	43.1	47.9	0.895	0.857
7.011	6.968	7.397	38.9	38.1	39	0.83	0.967
5.282	7.563	7.538	39.3	41.9	46.7	0.813	0.839
		1.925	53.5		60.8	0.81	
		7.141	38.9	35.8	40.7	0.82	0.938
7.172	7.586	7.544	37.3	34.7	37.2	0.76	0.854
5.011	5.886	6.26	36.3	34.9	40	0.77	0.833
		8.52	39.5	36.5	52.7	0.89	0.912
8.731	8.2	7.385				0.83	0.981
6.122	9.557	7.969	46	49.1	57.1	0.87	0.926
		6.476	28.1	38	40.5	0.86	0.899
8.216	9.06	8.544	32.5	41.6	43.4	0.79	0.878
7.147	6.996	7.89	29.5	36.7	34.3	0.87	0.914
		0.318	42.9	42.4	49.3	0.88	0.891
		11.2	37.2	37.9	40.7	0.87	0.796
7.312	7.047	7.582	41.9	43.5	48.8	0.744	0.916
8.308	8.775	9.6	33.4	38.5	38.7	0.794	0.845
4.631	8.787	8.209	38.8	36.7	39.8	0.887	0.765
		8.183	38.7		39.4	0.902	
9.461	8.862	10.067	45.7	45.9	55	0.852	0.888
5.95	10.65	8.37	33	35.7	40.4	0.889	0.847
6.872	8.659	8.453	40.8	38	39.1	0.85	0.959
8.835	5.174	8.434	43.2	43	46.8	0.73	0.887
9.501	7.863	9.432	40.1	39.9	41.7	0.778	0.941
7.11	7.282	8.097	35	39.9	44	0.934	0.859
4.493	7.246	6.961	42.2	43.7	41.9	0.804	0.841
9.143	8.495	8.986	33.9	33.7		0.84	0.909
		8.072	43.3		49	0.837	
7.421	7.573	7.949	38.7	34.2	44.2	0.78	0.862
4.136	7.727	6.79	47.8	51.8	57.2	0.78	0.81
		7.385	63.5	63	65.3	0.77	0.844
		7.341	36.2		32.3	0.8	
		8.115	47.7		59.8	0.75	
6.589	5.928	6.839	43.7	44	50.1	0.87	0.91
7.186	7.518	8.004	47.4	47.3	53.8	0.84	0.899

6.331	40.6	44.9	45	0.87	0.9
	41.3	44.9	40.4	0.78	0.79
12.3	44.2	42.9	42.8	0.87	0.832

RERMX	PaO2RST	PaO2LT	PaO2_MX	A_aDO2R	A_aDO2L	A_aDO2M	Vd_VtRS	Vd_VtLT
0.975	57.2		44.5	47		47	0.506	
0.981	81.7		63.7	12		37	0.759	
1.089	73.6	71.1	58.3	21	22	38	0.66	0.544
1.152	83.9	86.6	83.2	12	15	20	0.651	0.603
0.939	87.3	81.9	56.4	3	8	25	0.565	0.547
0.917	91.6	86.5	59.4	8	12	33	0.624	0.535
1.211	83.7	75.5	68.1	21	34	49	0.557	0.435
1.108	89	80.6	76.5	19	22	29	0.545	0.541
0.932	71.3	67.8	55.9	26	28	38	0.634	0.615
1.132	84.7	89.4	82.3	15	16	27	0.602	0.527
1.106	81.7	77.4	70.6	16	18	30	0.479	0.486
0.86	67.1		52.5	13		22	0.674	
1.04	74.6	74.4	76.1	25	34	30	0.648	0.492
1.25	90.9	91.1	97.7	8	15	16	0.581	0.43
1.08	84	83.9	89.7	17	21	18	0.609	0.492
1.11	74.8	74.1	64.6	28	32	33	0.548	0.465
1.14								
1.02	79.5	70	56.8	16	25	34	0.572	0.499
0.89	73	69.1	59.7	39	34	40	0.463	0.582
0.92	88	79	64.4	18	20	34	0.444	0.438
1.21	99.2	87.1	77.5	14	20	39	0.403	0.409
0.95	82.8	78.7	78.6	16	22	17	0.552	0.621
1.01	79.5	73.4	72.3	26	28	34	0.622	0.554
1.102	90	82.9	62.4	2	16	38	0.684	0.472
1.124	94	105	93.6	10	-5	15	0.625	0.481
0.952	67.6	71	75.5	35	29	29	0.629	0.45
1.012	80.2		88.2	24		20	0.545	
1.047	76.1	75.9	74.8	18	19	18	0.581	0.624
0.97	74	68.2	64	35	37	40	0.466	0.339
1.179	84	90	73.9	15	16	37	0.657	0.47
1.05	77.5	73.8	56.4	12	25	44	0.485	0.398
1.186	82.7	89.4	69.7	14	15	40	0.64	0.503
1.203	95	78.1	65.4	14	23	42	0.501	0.429
1.035	83.4	84.5	84	12	11	20	0.603	0.473
1.028	61.5	70.5		45	38		0.525	0.349
0.966	91.6		101.7	2		-8	0.497	
1.06	81.1	81.4	56.6	17	26	47	0.615	0.487
1.02	73.9	75.3	68.8	13	9	21	0.607	0.514
0.92	51.2	49.8	41.7	17	25	35	0.728	0.561
0.88	78.5		131.5	22		-23	0.599	
1.22	65.5		58.4	19		35	0.662	
1.22	72.6	66	57.4	24	32	45	0.614	0.424
1.14	66.5	63.1	53.3	24	30	43	0.498	0.41

1.03	87.2	80.6	65.9	14	17	37	0.575	0.441
1.11	81.2	78.6	88	15	14	21	0.599	0.496
1.01	69.7	68.6	84.2	28	28	20	0.495	0.435

VCO2RS	VCO2LT	VCO2MA	VERS	VELT	VEMX	VE_VCO2	VE_VCO2	VE_VCO2
225		688	9.875		24.8	43.889		36.047
152		510	13.45		25.5	88.487		50
196	410	867	11.425	16.6	27.7	58.291	40.488	31.949
200	364	909	11.675	17.9	34.9	58.375	49.176	38.394
240	332	864	9.4	12.6	25.8	39.167	37.952	29.861
189	342	888	11	16.4	33.4	58.201	47.953	37.613
175	429	1137	10.4	19.8	51.4	59.429	46.154	45.207
218	344	1158	12.525	16.4	44.1	57.454	47.674	38.083
179	246	396	9.775	12.8	18.2	54.609	52.033	45.96
176	464	1064	9.8	22.2	46.4	55.682	47.845	43.609
169	412	911	7.125	16.5	33	42.16	40.049	36.224
194		456	9.6		18.4	49.485		40.351
173	483	910	10.9	22.91	37.9	63.006	47.433	41.648
174	298	1200	9.6	13	50.3	55.172	43.624	41.917
209	349	1040	12.7	17	40.9	60.766	48.711	39.327
176	362	980	8.5	16	30.9	48.295	44.199	31.531
145	369	1150	11	21.8	53.9	75.862	59.079	46.87
212	413	808	9.3	14.5	24.4	43.868	35.109	30.198
222	311	493	12.7	16.9	24.4	57.207	54.341	49.493
176	374	646	8.4	13.8	22.3	47.727	36.898	34.52
337	498	1553	16.5	19.8	61.3	48.961	39.759	39.472
196	205	497	8.8	11	21	44.898	53.659	42.254
220	378	1154	13.5	19.3	45.5	61.364	51.058	39.428
160	602	1315	10.425	22.6	39.1	65.156	37.542	29.734
200	539	1803	13.775	23.3	60.1	68.875	43.228	33.333
244	388	1400	14.633	16.6	45.7	59.971	42.784	32.643
230	1548	1548	11.267		53.2	48.987		34.367
281	310	1326	12.675	15.5	39.1	45.107	50	29.487
240	487	1063	11.75	17.8	29.1	48.958	36.55	27.375
187	397	1006	11.533	17	41	61.674	42.821	40.755
203	465	1423	7.867	15.5	35.1	38.754	33.333	24.666
147	370	1629	8.8	16.1	55.2	59.864	43.514	33.886
253	671	2190	12.5	25.4	62	49.407	37.854	28.311
172	486	1240	8.85	18.2	39.1	51.453	37.449	31.532
226	577	2031	12.125	22.7	61.1	53.65	39.341	30.084
261		1268	10.35		41.5	39.655		32.729
159	370	1256	9.2	18.2	44	57.862	49.189	35.032
196	493	1078	9	16.9	29.9	45.918	34.28	27.737
126	372	815	6.3	11.6	20.2	50	31.183	24.785
170		777	10.1		29.2	59.412		37.58
213		1599	11.4		39.6	53.521		24.765
293	825	1965	15	28.1	53.5	51.195	34.061	27.226
292	456	1873	10.6	14.1	37	36.301	30.921	19.754

176	637	1532	8.8	21.9	45.9	50	34.38	29.961
169	454	1225	8.8	17.3	41.5	52.071	38.106	33.878
225	385	1154	8.7	13.7	35.5	38.667	35.584	30.763

PaETCO2l	PaETCO2l	PaETCO2l	VE_VO2R	VE_VO2L	VE_VO2M	HGBGM_	Hct	Hct_Hb
-2		2	41		35	13.7	40.442	2.952
12		5	66		49	13.7	39	2.847
5	3	-2	49	36	35	13.7	40.6	2.964
4	5	2	48	50	44	13.6	41.4	3.044
5	2	4	36	34	28	15.5	46.3	2.987
4	-2	0	48	38	35	15.3	46.9	3.065
2	-1	-2	46	41	55	15.2	45.2	2.974
-1	0	-14	50	43	42	14.5	42.6	2.938
5	4	5	49	45	43	12.6	37.195	2.952
8	3	3	46	46	49	13.2	38.966	2.952
5	3	3.7	34	34	40	11.8	36	3.051
9		10	40		35	14.6	45.6	3.123
9	0	3	51	44	43	15.9	48	3.019
4	0	3	43	37	53	13.9	41.7	3
7	1	-1	47	41	42	17.5	52.9	3.023
0	-3	0	43	40	35	15	45.9	3.06
			63	58	53	15.1	46	3.046
4	1	1	38	33	31	15.5	18.9	1.219
-5	5	5	49		44	15	41.8	2.787
-7	-1	-5	38	32	32	13.1	39.4	3.008
-8	-1	-3	43	36	48	10.9	34.5	3.165
5	9	6	39	48	40	12.9	40.5	3.14
8	5	4	53	41	43	14.8	43.9	2.966
2	0	-2	48	34	33	14.9	42.4	2.846
4	3	-1	55	37	37	14.7	44.5	3.027
8	1	-3	53	33	31	16.8	49.9	2.97
6.2		-2	44		35	13.2	39.8	3.015
11	9	3	38	44	31	14.4	42.508	2.952
-1	-5	-8	44	31	27	17.1	50.479	2.952
8	1	1	52	41	48	13.5	38.3	2.837
3	-3	-1	28	30	26	14.9	43.5	2.919
4	2	1	47	41	40	15.4	43.7	2.952
2	0	-8	46	33	34	15.4	43.7	2.952
4	-1	-6	41	31	33	14.1	41	2.908
4	-3		45	36	31	14.7	43.5	2.959
9		6	33		32	13.4	40	2.985
7	1	2	45	42	37	15.8	47	2.975
2	0	-4	46	28	28	14.5	43.9	3.028
11	6	1	38	26	23	18.9	62.5	3.307
5		0	48		33	17		
0		-3	40		30	16.5	52.9	3.206
4	-1	-5	44	31	33	16.7	47.5	2.844
0	-4	-16	31	28	22	16.1	46.7	2.901

7	2	-1	44	31	31	14	41.9	2.993
5	1	-4	41	30	39	16.5	49.3	2.988
3	-1	-4	34	30	31	15.9	48	3.019

COHGB	METHGB	PLATELE	ALBUMIN	GLOBULI	TTL_PRO	GOT	GPT	BIL_D
2	0.3		4.4	3.3	7.7	18	11	0.2
0.3	0.3	16.6	3.8	3.6	7.4	37		0.3
2.8	0.3	22.2	4.2	2.6	6.8	17	12	0.2
1.5	0.3		4	3.8	7.8	16	14	0.2
1	0	25.9	4.7	2.9	7.6	33	16	0.1
2.1	0.2	19.3	4.2	2.7	6.9	13	7	0.2
0.8	0.1	24.1	3.8	2.6	6.4	59	76	0.2
1.2	0.3	17.9	4.3	2.9	7.2	21	17	0.2
2.2	0.2		3.6	3.4	7	23	16	0.1
2.7	0.3	31.3	4.5	3.5	8			0.2
0	0	27.8	4	2.5	6.5	24	12	0.1
1.4	0.3	36	4.3	3.9	8.2	79	47	0.1
1.5	0.3	14.8						
2.2	0.3	22.2	4.1	3.3	7.4	20	9	0.2
1.2	0.3	23.5				15	7	0.1
2	0.3	38.4	4	3.3	7.3	48	64	0.1
1		16						
0.9	0.3	25.8				21	15	0.2
1.3	0.3	27.1	3.9	2.6	6.5	22	34	0.7
0.1	0.2	23	3.1	2.8		24	25	0.2
0.1	0.2	31	4.2		7.3	13	5	2
0.9	0.3	31.1	4.2	3.3	7.5	36	26	0.2
0.8	0.3	19	4.1	2.9	7	17	14	0.2
1.5	0.2	19	4.5	2.7	7.2	23	11	0.2
0.1	0.1	21.7	3.8	2.7	6.5	15	11	0.2
1.1	0.3	29.3	4.2	2.3	6.5	74	67	0.3
0.6	0.2		4.1	2.7	6.8	24	28	0.2
0.5	0.3		3.8	1.9	5.7	16	12	0.2
1.4	0.3	16.5	4.1	2.6	6.7	25	18	0.2
2	0.2	19.1	4.2	2.4	6.6	25	16	0.2
1.6	0.3	23.1	4.4	2.6	7	22	17	0.2
2.5	0.3	27.2	3.1	4.7	7.8	34	25	0.2
1.2	0.2	27.2	4.6	3.3	7.9	24	18	0.3
1	0.3	24	4.7	3.1	7.8	33	15	0.1
0.9	0.1	22.7	4.1	2.7	6.8	16	11	0.3
		27.3	4.3	3.4	7.7	16	8	0.2
1.7	0.3	17.4	4.2	3	7.2	20	12	0.2
1.2	0.3	25.6	4.3		7.6	21	17	0.2
4.7	0.3	18.1	4	3.1	7.1	24	12	0.2
1.1	0.2							
		21.6	4.2		7.5	23	14	0.2
1	0.3	18.5				19	14	0.1
0.3	0.3		5		7.4			

2.2	0.3	21.3	4.3	2.5	6.8	43	49	0.2
0.8	0.3	26.7	4.4	3.1	7.1	18	13	0.2
1.4	0.3	20.1	4.4	2.5	6.9	27	28	0.2

Cr	Na	K	Cl	SUGAR	CHOL	TG	veo2mx_lt	PO2mx_rs
1.1	139	4.2	105	110	190	110		-12.7
0.8	139	4.4	109	105	170	104		-18
1	142	4.5	111	90	210	81	0.979	-15.3
0.7	139	5.1	112	96	140	61	0.892	-0.7
1	137	5.2	103	92	214	146	0.821	-30.9
0.9	140	4.7	111	98	162	65	0.9	-32.2
1.1	143	3.9	114	116	177	91	1.324	-15.6
1.3	145	3.9	112	107	242	99	0.991	-12.5
1.2	141	4.2	106	92	253	89	0.96	-15.4
	141	4.6		90	139	49	1.067	-2.4
1	142	4.1	108	98	176	53	1.192	-11.1
1.2	136		101	67	213	51		-14.6
							0.975	1.5
1.1	139	4.1	109	85	174	83	1.41	6.8
0.9	138	4.1	115	103	93	141	1.044	5.7
1.4	140	4.5		91	178		0.867	-10.2
							0.92	
1.2	141	3.6	104	105	203		0.944	-22.7
1.1	135	4.2	103	114	193		0.004	-13.3
1	141	4.6		103	184		0.983	-23.6
0.9	139	3	108	96	141	44	1.311	-21.7
1.1				95	243	89	0.84	-4.2
1	141	4	108	89	242	235	1.047	-7.2
1.2	140	5	107	115	195	179	0.953	-27.6
1.1	140	3.9	109	86	169	73	1.026	-0.4
0.9	144	4	109	87	193	110	0.95	7.9
1.1	141	4.6	107	152	165	140		8
1.1	141	4.4	111	95	194	114	0.695	-1.3
1	139	4	109	86	144	54	0.858	-10
1.3	143	4.8	110	96	182	138	1.171	-10.1
1.1	140	4	108	122	225	95	0.876	-21.1
0.9	135	4.4		97	107	57	0.981	-13
0.9	140	4.4	110	121	165	47	1.047	-29.6
1.3	141	4.5	108	99	195	63	1.037	0.6
0.9	142	4	114	89	156	86	0.865	
1.6				103	235			10.1
1.1	141	4.4	111	85	175	71	0.876	-24.5
1	141	4.2	106	90	158	86	1.021	-5.1
1	139	5	98	86	187	60	0.87	-9.5
1.4	146	2.6	91	116	169	90		-7.1
1.2	140	4.7	107		110	96	1.074	-15.2
1	142	4.4			224	82	0.808	-13.2

1	144	4.3	111	79	153	46	0.995	-21.3
1.7	141	4.3	104	91	191	202	1.289	6.8
1	143	4.2	109	114	205	235	1.051	14.5

PCO2mx_:	MVV	FEV1_35	FEV1_20+	FEV1_40	FEV1	VERS_M\	VELT_M\	VEMX_M
12.9	30	30.8	37.6	35.2	0.88	0.329		0.827
3.6	30.3	36.4	40.8	41.6	1.04	0.444		0.842
9.2	18.4	30.8	37.6	35.2	0.88	0.621	0.902	1.505
4.6	30.6	45.5	46	52	1.3	0.382	0.585	1.141
9.2	17.2	25.55	34.6	29.2	0.73	0.547	0.733	1.5
9.5	17	44.1	45.2	50.4	1.26	0.647	0.965	1.965
-0.6	51.1	63.35	56.2	72.4	1.81	0.204	0.387	1.006
10.3	46.6	60.9	54.8	69.6	1.74	0.269	0.352	0.946
4.7	32.2	32.2	38.4	36.8	0.92	0.304	0.398	0.565
0.1	57.05	46.9	46.8	53.6	1.34	0.172	0.389	0.813
7.4	39.5	41.65	43.8	47.6	1.19	0.18	0.418	0.835
7.3	11.1	10.5	26	12	0.3	0.865		1.658
1.8	33.25	33.25	39	38	0.95	0.328	0.689	1.14
-0.1	51.45	51.45	49.4	58.8	1.47	0.187	0.253	0.978
3.7	30.8	34.65	39.8	39.6	0.99	0.412	0.552	1.328
13.2	35	26.25	35	30	0.75	0.243	0.457	0.883
	98	87.5	70	100	2.5	0.112	0.222	0.55
11.1	16.88	21.7	32.4	24.8	0.62	0.551	0.859	1.445
12.4	25.66	18.9	30.8	21.6	0.54	0.495	0.659	0.951
10.9	25.4	28	36	32	0.8	0.331	0.543	0.878
4.8	81	95.9	74.8	109.6	2.74	0.204	0.244	0.757
6.4	21	25.55	34.6	29.2	0.73	0.419	0.524	1
3.5	44.29	30.1	37.2	34.4	0.86	0.305	0.436	1.027
6.9	30.7	39.9	42.8	45.6	1.14	0.34	0.736	1.274
5.3	55	49.7	48.4	56.8	1.42	0.25	0.424	1.093
1	28.8	30.8	37.6	35.2	0.88	0.508	0.576	1.587
0.7	45.9	65.1	57.2	74.4	1.86	0.245		1.159
9.3	20.7	32.2	38.4	36.8	0.92	0.612	0.749	1.889
7.4	18.4	27.65	35.8	31.6	0.79	0.639	0.967	1.582
-1.7	25.2	38.85	42.2	44.4	1.11	0.458	0.675	1.627
3.6	26.3	37.8	41.6	43.2	1.08	0.299	0.589	1.335
1.6	54.6	63.7	56.4	72.8	1.82	0.161	0.295	1.011
9	48.1	65.1	57.2	74.4	1.86	0.26	0.528	1.289
-0.3	37	46.2	46.4	52.8	1.32	0.239	0.492	1.057
	45	58.1	53.2	66.4	1.66	0.269	0.504	1.358
5.7	49.4	36.75	41	42	1.05	0.21		0.84
5.5	34	33.6	39.2	38.4	0.96	0.271	0.535	1.294
9.4	23	22.4	32.8	25.6	0.64	0.391	0.735	1.3
1.8	30	31.5	38	36	0.9	0.21	0.387	0.673
-3.9	41	39.55	42.6	45.2	1.13	0.246		0.712
12.1	32.2	40.6	43.2	46.4	1.16	0.354		1.23
6.4	30.63	29.4	36.8	33.6	0.84	0.49	0.917	1.747
6.4	64.8	52.15	49.8	59.6	1.49	0.164	0.218	0.571

4.4	28.2	33.6	39.2	38.4	0.96	0.312	0.777	1.628
-0.9	28	36.05	40.6	41.2	1.03	0.314	0.618	1.482
-1.4	28	38.5	42	44	1.1	0.311	0.489	1.268

BrRes
0.173
0.158
-0.505
-0.141
-0.5
-0.965
-0.006
0.054
0.435
0.187
0.165
-0.658
-0.14
0.022
-0.328
0.117
0.45
-0.445
0.049
0.122
0.243
0
-0.027
-0.274
-0.093
-0.587
-0.159
-0.889
-0.582
-0.627
-0.335
-0.011
-0.289
-0.057
-0.358
0.16
-0.294
-0.3
0.327
0.288
-0.23
-0.747
0.429

-0.628
-0.482
-0.268
